# Catalytic Hydrogenation Dominated by Concerted Hydrogen Tunneling at Room Temperature

**DOI:** 10.1021/acscentsci.5c00943

**Published:** 2025-09-26

**Authors:** Qingyuan Wu, Pengxin Liu, Xia-Guang Zhang, Cheng Fan, Ziwen Chen, Ruixuan Qin, Yi Qin Gao, Yi Zhao, Nanfeng Zheng

**Affiliations:** † New Cornerstone Science Laboratory, State Key Laboratory for Physical Chemistry of Solid Surfaces, iChEM, and National & Local Joint Engineering Research Center for Preparation Technology of Nanomaterials, College of Chemistry and Chemical Engineering, 12466 Xiamen University, Xiamen 361005, China; ‡ School of Physical Science and Technology, 387433 ShanghaiTech University, Shanghai 201210, China; § Key Laboratory of Green Chemical Media and Reactions, Ministry of Education, Collaborative Innovation Center of Henan Province for Green Manufacturing of Fine Chemicals, School of Chemistry and Chemical Engineering, 66519 Henan Normal University, Xinxiang 453007, China; ∥ Institute of Theoretical and Computational Chemistry, College of Chemistry and Molecular Engineering, 12465 Peking University, Beijing 100871, China; ⊥ Innovation Laboratory for Sciences and Technologies of Energy Materials of Fujian Province (IKKEM), Xiamen 361102, China; # Changping Laboratory, Beijing 102200, China

## Abstract

Tunneling control of chemical reactions is treasured as the third reactivity paradigm, next to kinetic and thermodynamic control. However, reports on the successful observation and mechanistic insight into quantum tunneling in conventional heterogeneous catalysis are limited. By using an atomically dispersed palladium catalyst, we now demonstrate room-temperature catalytic hydrogenation dominated by concerted triple hydrogen tunneling. While a large kinetic isotope effect value of ∼2440 is observed in the benzyl aldehyde hydrogenation when both H_2_ and solvent (CH_3_OH) are deuterated, the use of protic solvent is important to achieve enhanced catalysis. Systematic investigations reveal that, with a protic solvent molecule situated between the catalytic site and aldehyde, the formation of a local hydrogen bond network helps to induce the concerted triple hydrogen tunneling, namely, that two protons transfer from the ligand on the catalytic site to the mediated solvent and the oxygen of C=O on aldehyde, respectively, and the other transfers from Pd on the catalytic site to the carbon of C=O on aldehyde. With the width and height of the potential energy barrier alterable by protic solvents, the hydrogen tunneling probability can be regulated by solvents. Furthermore, far-infrared irradiation is found to enhance the hydrogenation rate.

## Graphical abstract

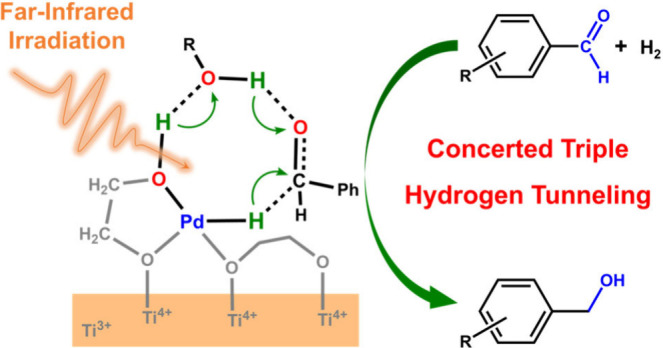

## Introduction

Quantum tunneling, which refers to the ability of a microscopic particle to penetrate a potential energy barrier when the total energy is less than the potential energy barrier,^1,2^ is ubiquitous in astrochemistry,^3^ biochemistry,^4–8^ molecular reactions,^9–14^ organometallic chemistry,^15–18^ homogeneous catalysis,^19–21^ and surface science.^22–26^ The tunneling occurs when the transfer distance of the microscopic particle is on the order of its *de Broglie* wavelength, and its transmission probability in terms of the Wentzel–Kramers–Brillouin approximation brings to light that a lighter particle tunnels more easily across a narrow and low barrier.^1,2,27,28^ Benefited from a great number of experimental findings and theoretical insights, tunneling control has emerged as a strategy different from kinetic and thermodynamic methods to tailor the reaction rate, selectivity, and even the mechanism of chemical transformations.^9–12,29–32^ While quantum tunneling has been extensively reported in homogeneous catalysis, there are few reports on quantum tunneling in heterogeneous catalysis.^22,33,34^ This is partly because heterogeneous catalytic processes often occur at high temperatures, where the tunneling contribution is negligible compared with the thermodynamic contribution,^3^ and also partly because of the complexity of heterogeneous catalysts making it challenging to distinguish the tunneling contribution.^35–37^ In this regard, heterogeneous model catalysts with uniform and well-defined catalytic sites are highly desirable for investigating tunneling effects in heterogeneous catalysis.^38–44^ With no doubt, atomically dispersed metal catalysts represent such a class of catalysts for the investigation owing to their maximum atom efficiency and well-defined active sites.^45–48^

Herein, we demonstrate a room-temperature heterogeneous catalytic hydrogenation process dominated by concerted triple H-tunneling on an atomically dispersed Pd catalyst. During the hydrogenation of benzaldehyde to benzyl alcohol, H_2_ was activated over the catalyst in a heterolytic manner. An extremely large kinetic isotope effect (KIE) of ∼2440 was observed at room temperature when H_2_ and CH_3_OH were replaced by deuterated ones. Detailed experimental and theoretical analyses demonstrate that such a large KIE is caused by an unprecedented hydrogenation mechanism dominated by concerted triple H-tunneling involving one hydride (Pd–H) and two protons (O–H) within the hydrogen bond network. Moreover, the replacement of CH_3_OH by other protic solvents can regulate the tunneling probability by changing the height and width of the energy barrier to alter the hydrogenation rate. The catalytic activity can be further promoted by far-infrared (FIR) irradiation to excite reactants to higher vibrational energy levels.

## Results and Discussion

An atomically dispersed Pd catalyst (Pd_1_/TiO_2_), in which Pd was supported atomically on ethylene glycolate (EG)-stabilized TiO_2_(B) nanosheets, was prepared by a photochemical method previously reported by our group^43^ and employed as the model catalyst to investigate the possible involvement of the H-tunneling process in catalytic hydrogenation. The catalyst was first evaluated in the catalytic hydrogenation of benzaldehyde in CH_3_OH at 303 K (Figure 1a). As shown in Figure 1b, an excellent catalytic performance was observed with benzaldehyde being completely converted to benzyl alcohol in ∼60 min. Even when the reaction time was extended to 120 min, no overhydrogenated toluene byproduct was detected by ^1^H NMR (Figure S1). In comparison, much lower activity was observed when either Pd/C or H_2_PdCl_4_ was used as a catalyst under the same reaction conditions (Figure S2). Moreover, no significant decrease in catalytic activity was observed over six recycling tests, demonstrating the excellent stability of the catalyst (Figure S3). In addition to benzaldehyde, the catalyst also efficiently catalyzed the hydrogenation of various other aromatic aldehydes (Table S1).

**Figure 1:**
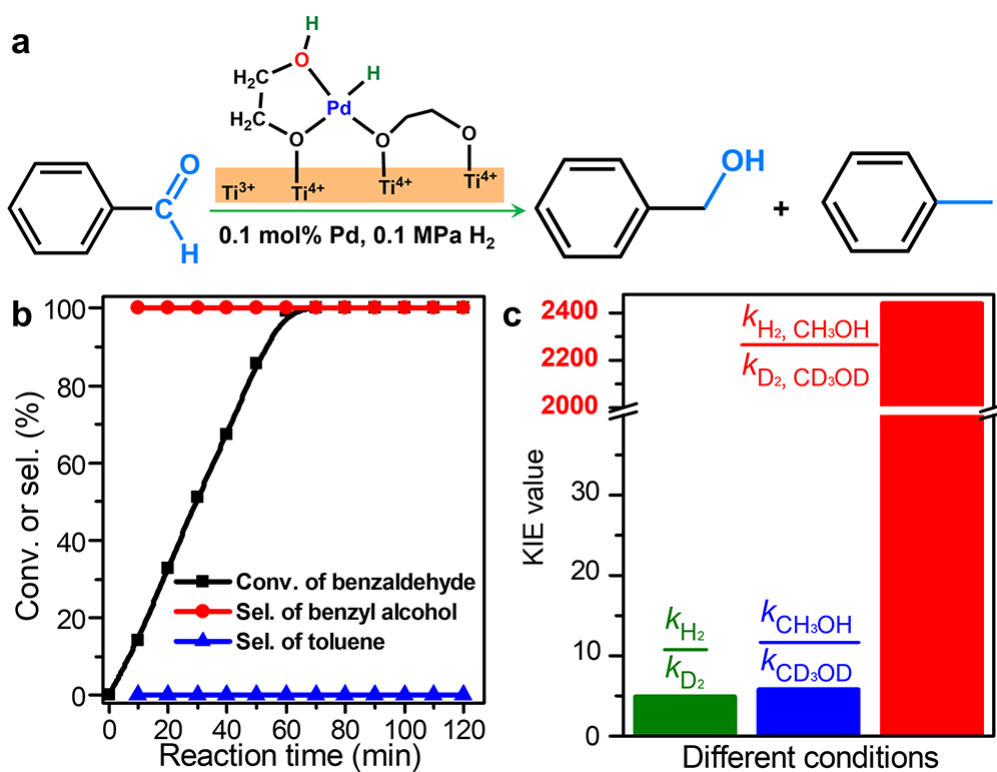
Concerted multiple H-tunneling in CH_3_OH. (a) Hydrogenation of benzaldehyde catalyzed by Pd_1_/TiO_2_. (b) Reaction performance of Pd_1_/TiO_2_ in benzaldehyde hydrogenation. (c) KIE values of *k*_H2_/*k*_D2_, *k*_CH3OH_/*k*_CD3OD_, and *k*_H2, CH3OH_/*k*_D2, CD3OD_ observed for Pd_1_/TiO_2_ in benzaldehyde hydrogenation at 303 K.

The excellent catalytic performance motivated us to further understand the transfer behavior of the active H species in the rate-determining step (RDS) of benzaldehyde hydrogenation. KIE experiments were first performed using three sets of conditions in which H_2_ or/and CH_3_OH were replaced by D_2_ and CD_3_OD, respectively (Figures 1c and S4).^43^ While the reaction was determined to be zero order, the KIE (*k*_H2_/*k*_D2_) value in which only H_2_ was replaced by D_2_ was measured at 303 K to be 4.94. Considering the heterolytic activation of hydrogen molecules at the catalytic Pd sites, such a primary KIE indicated that O–D on the EG ligand at the catalytic site was directly involved in the RDS of the reaction.^49,50^ When H_2_ was used with CD_3_OD as the solvent, the KIE (*k*_CH3OH_/*k*_CD3OD_) value was measured to be 5.74, suggesting the direct involvement of the solvent O–D in the hydrogenation as well as O–D bond cleavage in the RDS. Surprisingly, when both H_2_ and CH_3_OH were replaced by D_2_ and CD_3_OD, respectively, an unexpectedly large KIE (*k*_H2, CH3OH_/*k*_D2, CD3OD_) value of ∼2440 was obtained. Such a value is much larger than that of KIE (*k*_H2_/*k*_D2_) or KIE (*k*_CH3OH_/*k*_CD3OD_) and even the product of these two. This result suggests that both O–H at the catalytic center and O–H on the CH_3_OH solvent should be transferred via a quantum tunneling mechanism, probably in a concerted way.^51–53^

Density functional theory (DFT) calculations were conducted to investigate the potential reaction mechanism of quantum H-tunneling with a large KIE. Based on the experimental results and previous studies,^43^ a cluster model was constructed to reflect the structure of the active site of the catalyst. In the constructed model, while a Pd atom was bound to a titanium oxide cluster by an EG ligand, the dangling bonds of the oxygen atoms of the cluster were saturated with hydrogen atoms (Figure S5). The energy profile of H_2_ activation at the Pd site was calculated before proceeding to hydrogenation (Figure S6). Once adsorbed on Pd, H_2_ was readily split into two H atoms by heterolytic activation. The step was calculated to be exothermic by ∼102.3 kJ/mol with a barrier of ∼66.7 kJ/mol. Natural population analysis indicated that one of the H atoms moved to a nearby oxygen on EG to yield O–H^δ+^, leaving the other H atom on Pd as H^δ−^. It is noteworthy that the reaction barriers and charge distribution agree well with previous DFT results using periodic boundary conditions,^43^ suggesting that this structure model should be suitable for studying the reaction system.

As the O–D of the EG ligand and CH_3_OH were directly involved in the RDS of benzaldehyde hydrogenation, we constructed the reaction model with a methanol molecule directly participating in the hydrogenation reaction (Figure S7). The model accounts for the possible reaction pathways for the hydrogenation of benzaldehyde, which include the concerted transfer of two O–H and one Pd–H as well as the stepwise mechanism. The reaction barriers and KIE values were calculated using the transition state theory and Eckart barrier model, respectively.^54^ The mechanisms involving stepwise H transfer were first considered. However, no reasonable KIE (*k*_H2, CH3OH_/*k*_D2, CD3OD_) values were obtained (Table S2). Therefore, the concerted tunneling of H on O–H and Pd–H at the Pd–O interface and H on CH_3_OH was considered. While the reaction barrier for the concerted mechanism was calculated to be ∼112.9 kJ/mol, the reaction was an overall exothermic process with an energy of ∼24.2 kJ/mol (Figure S7). KIE values at 298 K were calculated to be 60.0, 7.0, and 10.5 for *k*_H2, CH3OH_/*k*_D2, CD3OD_, *k*_H2_/*k*_D2_, and *k*_CH3OH_/*k*_CD3OD_, respectively (Table S3). Although the absolute values of the calculated KIE values were different from the experimental measurements, they followed the same trend, suggesting that the concerted triple H-tunneling mechanism might be more appropriate for the hydrogenation. To further support the concerted triple H-tunneling mechanism, the temperature-dependent KIE values of *k*_H2_/*k*_D2_, *k*_CH3OH_/*k*_CD3OD_, and *k*_H2, CH3OH_/*k*_D2, CD3OD_ were also calculated and revealed to significantly increase as the temperature decreases, especially the value of *k*_H2, CH3OH_/*k*_D2, CD3OD_ (Table S3, Figures 2a and S8).

**Figure 2:**
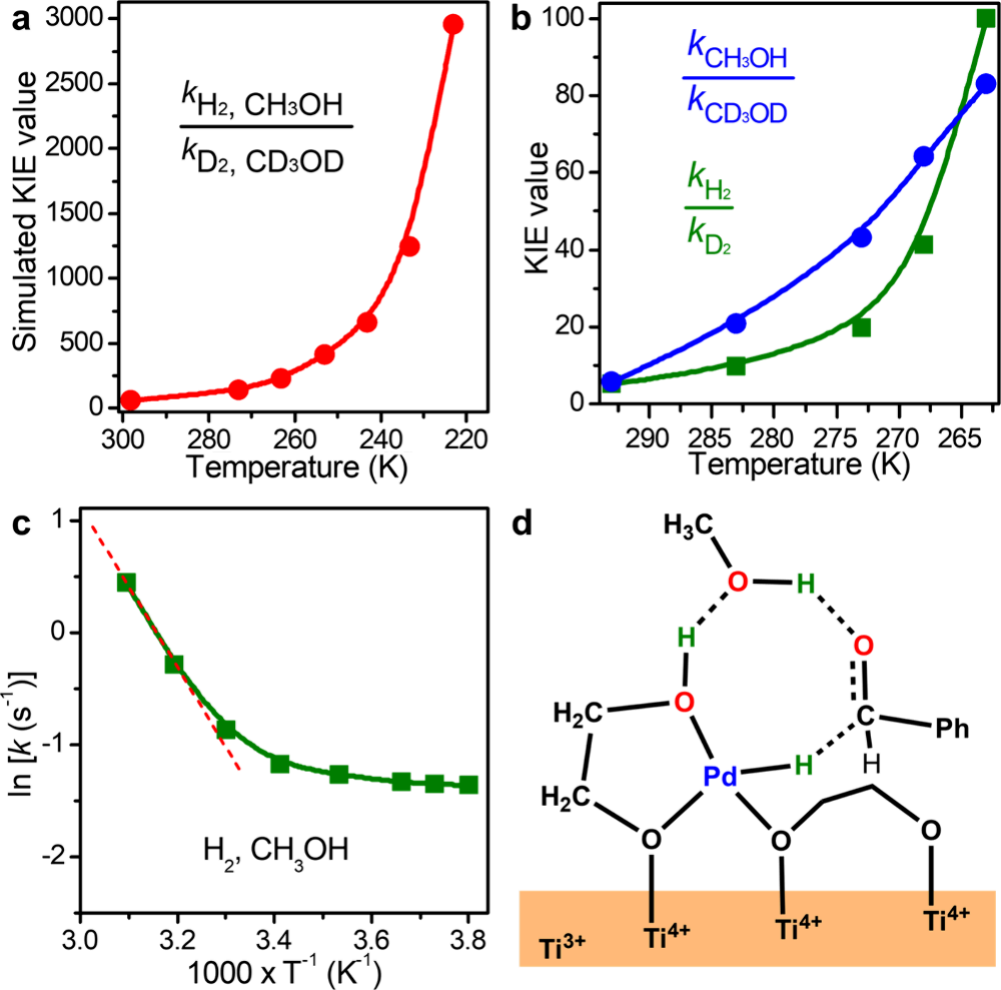
Concerted triple H-tunneling mechanism in CH_3_OH. (a) Simulated KIE (*k*_H2, CH3OH_/*k*_D2, CD3OD_) at different temperatures. The calculated absolute rate constants are summarized in Table S4. (b) Measured KIE values of *k*_H2_/*k*_D2_ and *k*_CH3OH_/*k*_CD3OD_ at different temperatures. (c) The Arrhenius plots by using H_2_ and CH_3_OH. The red dashed line indicates a trend dominated by thermodynamics. (d) The schematic diagram of the initial reaction state in CH_3_OH.

In order to gain a dynamically mechanistic understanding of the large KIE in concerted triple H-tunneling, path-integral molecular dynamics (PIMD) simulations were conducted. Utilizing H_2_ and CH_3_OH, the simulated free energy energies of PIMD and classical molecular dynamics (CMD) were determined to be 64.9 and 87.3 kJ/mol, respectively (Figures S9 and S10). This substantial reduction in the energy barrier can be attributed to the quantum mechanical behavior of hydrogen atoms with tunneling playing a dominant role. Furthermore, isotopic substitution studies revealed distinct energy landscapes for the two proton transfers involved in the reaction pathway. Deuteration of H_2_ resulted in only a modest increase of 4.1 kJ/mol in the energy barrier (Figures S9 and S11), whereas deuteration of CH_3_OH led to a much greater increase of 16.5 kJ/mol (Figures S9 and S12). The results support the hypothesis that proton transfer from the solvent is the dominant pathway in the concerted triple hydrogen tunneling mechanism. The small discrepancy between the energy barriers of only CH_3_OH deuteration (81.4 kJ/mol, Figure S12) and full deuteration (83.4 kJ/mol, Figure S13) systems also supports this conclusion. This mechanistic hierarchy provides an explanation for the initial slow reaction stage in the CH_3_OH-deuterated system (H_2_, CD_3_OD), as shown in Figure S4.

The initial kinetic impediment can be attributed to the inefficient transfer of deuterium from CD_3_OD to the reactant. As the reaction proceeds, hydrogen–deuterium exchange converts CD_3_OD to CD_3_OH, resulting in a gradual acceleration of the reaction until the rate is similar to that of the hydrogen-deuterated (D_2_, CH_3_OH) systems. The computed quantum free energy barriers can be further used to estimate the reaction rates with the quantum transition-state theory (QTST) method.^55,56^ The computed KIEs for D_2_/CH_3_OH, H_2_/CD_3_OD, and D_2_/CD_3_OD are 6.27, 910.5, and 2335.0, respectively. These results display good agreement with the experiments. In addition, as the transfer of hydride occurred spontaneously once the other two proton transfers had been completed, the hydride transfer from Pd to the carbonyl carbon played a minor role in the concerted H-tunneling process.

Experimentally, we also measured the temperature-dependent KIE values of *k*_H2_/*k*_D2_ and *k*_CH3OH_/*k*_CD3OD_. The KIE (*k*_H2_/*k*_D2_) values at 293, 283, 273, 268, and 263 K were measured to be 5.14, 9.81, 19.8, 41.3, and 100, respectively (Figures 2b and S14), suggesting that the H transfer from the interfacial O–H and Pd–H sites involved a non-negligible tunneling contribution. Similarly, the KIE (*k*_CH3OH_/*k*_CD3OD_) values at 293, 283, 273, 268, and 263 K were measured to be 5.78, 20.8, 43.2, 64.1, and 82.9, respectively (Figures 2b and S15), also indicating the presence of considerable tunneling contribution in the H transfer involving solvent O–H. Other important experimental evidence to assess the contribution of quantum tunneling is to fit kinetic data with an Arrhenius expression. With the use of H_2_ and CH_3_OH, less temperature-independent reaction rates were observed at temperatures lower than 303 K (Figure 2c), leading to a slope tending to zero. These results demonstrate that the H tunneling already makes a significant contribution to the catalytic hydrogenation at room temperature. On the contrary, due to the negligible tunneling contribution, no detectable slope change was observed when Pd/C with Pd nanoparticles on a carbon support was used as the catalyst (Figure S16). Based on both the experimental and computational results, the hydrogen bond network formed by the catalytic site, solvent CH_3_OH molecule, and benzaldehyde should make a significant contribution to the concerted H-tunneling mechanism (Figure 2d).

As the concerted triple H-tunneling mechanism involves hydrogen bonds intermediated by the solvent molecules, with the tunneling of solvent protons to reactant being the more dominant process, the reaction rate and KIE should be regulatable by the use of different protic solvents. With the use of H_2_O as the solvent, the hydrogenation catalyzed by Pd_1_/TiO_2_ was calculated to be exothermic (14.7 kJ/mol) with a free energy barrier of 102.6 kJ/mol (Figure S17), ∼10 kJ/mol lower than that of CH_3_OH. As expected by the tunneling mechanism, the lower barrier height and the same tunnel breadth (Table S5) with the use of H_2_O should lead to faster reaction rates and smaller KIE values than those with CH_3_OH. Indeed, the calculated *k*_H2_/*k*_D2_, *k*_H2O_/*k*_D2O_, and *k*_H2, H2O_/*k*_D2, D2O_ values in H_2_O are of similar magnitude to those in CH_3_OH (Table S6 and Figure S18), suggesting the same reaction mechanism in both solvent systems.

As encouraged by these calculations, we experimentally measured the catalytic activities with the use of different protic solvents. Since the H transfer of O–H on solvent is dominated by tunneling, different properties of O–H (e.g., bond length and energy) should influence the relevant tunneling width and barrier height, resulting in different H-tunneling probabilities (Figure 3a).^27^ Therefore, we measured the turnover frequency (TOF) numbers of benzaldehyde hydrogenation in different protic solvents. The highest TOF was obtained with the use of H_2_O as the solvent, followed by CH_3_OH, *tert*-butanol, and cyclohexanol (Figure 3b). The temperature-dependent KIE values of the reaction in H_2_O were measured to further verify the concerted triple H-tunneling mechanism (Figure S19). The KIE values of *k*_H2, H2O_/*k*_D2, D2O_ increased significantly as the temperature decreased; in particular, a large value of 83.45 was measured at low temperature (274 K). The large KIE value also indicated that the transfer of both H at the Pd–O interface site and O–H of H_2_O underwent the concerted quantum tunneling mechanism. Furthermore, the Arrhenius curve also suggested a concerted triple H-tunneling mechanism. While the reaction rates did not show a significant temperature dependence when H_2_ and H_2_O were used (Figure 3c), the temperature dependence was more significant when D_2_ and D_2_O were used. As the highest efficiency is achieved in H_2_O, introducing H_2_O to the reactions in other solvent systems is expected to improve the catalytic rate. Experimentally, adding H_2_O to the reaction in CH_3_OH led to an enhanced hydrogenation rate and reached the same level when the volume ratio of H_2_O to CH_3_OH was 2 (Figure S20). The importance of the hydrogen bond network in the concerted triple H-tunneling mechanism was also verified by using anhydrous tetrahydrofuran (THF) as a control solvent. In sharp contrast to reactions in protic solvents, the conversion of benzaldehyde in THF was far lower than those in H_2_O at the same conditions (Figure 3d).

**Figure 3:**
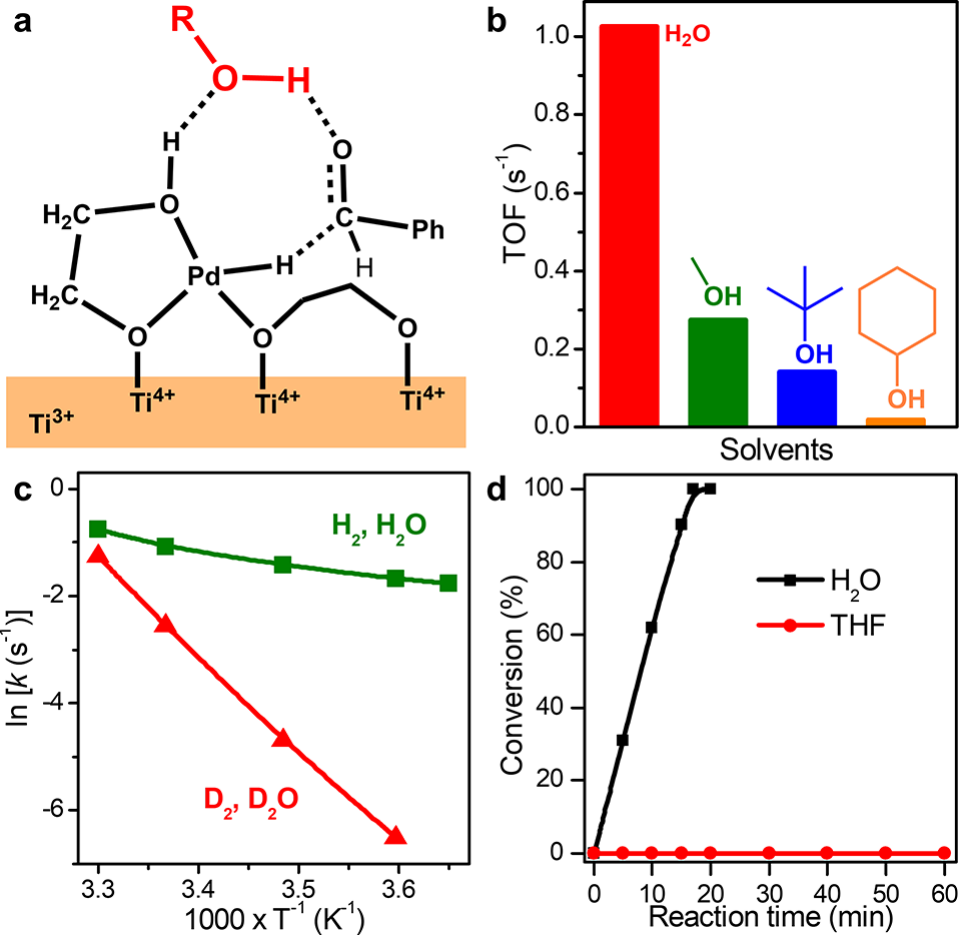
Tunneling probability regulated by protic solvents. (a) The schematic diagram of the initial reaction state in different types of protic solvent; R was H or alkyl. (b) The catalytic performances of Pd_1_/TiO_2_ in H_2_O, CH_3_OH, *tert*-butanol, and cyclohexanol. (c) The Arrhenius plots by using (H_2_, H_2_O) and (H_2_, D_2_O). (d) Hydrogenation of benzaldehyde catalyzed by Pd_1_/TiO_2_ in H_2_O and anhydrous THF.

With the involvement of the local hydrogen bond network in the tunneling mechanism, we suggest that the H-tunneling probability may be enhanced by exciting the hydrogen bond network to a higher energy vibrational level, potentially leading to a narrower barrier width (ω) and a lower barrier height (*V*_0_ – *E*) (Figure 4a).^57^ To explore this hypothesis, we applied far-infrared (FIR) irradiation, electrically generated by a graphene film, to the hydrogenation reaction (Figure S21).^58^ The terahertz bands provided by the FIR irradiation are expected to excite intermolecular vibrations, including hydrogen bond stretching and torsion modes, of the hydrogen bond network formed in H_2_O or CH_3_OH (Figure S22).^59^ As expected, the FIR irradiation enhanced the conversion rate of benzaldehyde in H_2_O with enhancement factors of 1.12, 1.23, and 1.30 observed at irradiation powers of 1, 3, and 5 W, respectively (Figure 4b). Similarly, the hydrogenation of benzaldehyde in CH_3_OH was also promoted under FIR irradiation with an enhancement factor of 1.22 at 5 W (Figure S23). In contrast, negligible changes in catalytic activity were observed when Pd/C was used as the catalyst under FIR irradiation (Figure S24). To rule out any potential thermal effects from the low-power FIR irradiation, a fast-circulating cooling water system was employed to maintain a constant reaction temperature of 0 °C during the hydrogenation catalysis. Moreover, as shown in Figure 2c, the reaction rate exhibits a minimal temperature dependence near 0 °C. The observed enhancement in activity induced by FIR irradiation warrants further investigation.

**Figure 4:**
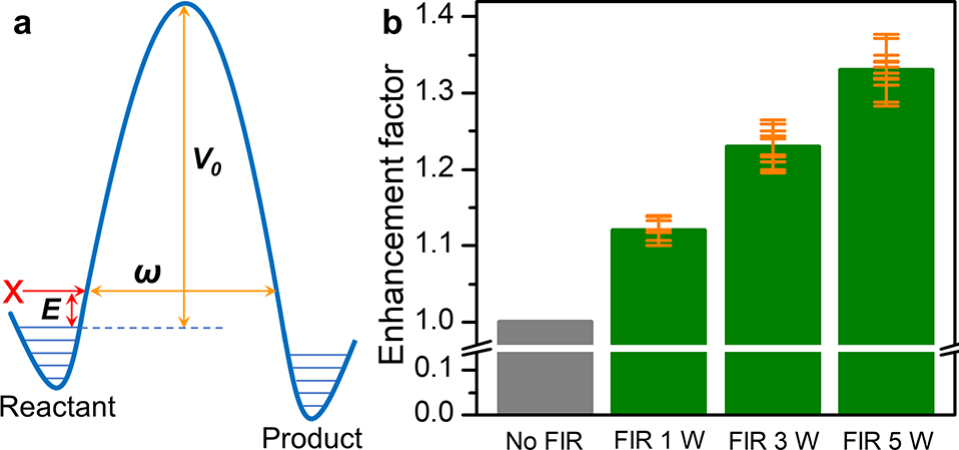
Tunneling probability improved by far-infrared irradiation. (a) The schematic diagram of increased energy level with narrower barrier width (ω) and lower barrier height (*V*_0_ – *E*). (b) Enhanced performance of Pd_1_/TiO_2_ in the hydrogenation of benzaldehyde carried out in H_2_O under FIR irradiation of varying power, generated by a graphene film.

## Conclusion

In conclusion, an atomically dispersed Pd catalyst on ultrathin TiO_2_ nanosheets is employed to gain insight into the mechanism of tunneling hydrogen transfer in catalytic hydrogenation. Concerted multiple H-tunneling was discovered in protic solvent rather than in protic-free solvent by systematic kinetic isotope effects and Arrhenius plots. Tunneling-controlled catalytic hydrogenation of benzaldehyde occurred in CH_3_OH at room temperature and in H_2_O at low temperature. DFT and tunneling transmission probability calculations also visualized the reaction mechanisms of concerted triple H-tunneling in benzaldehyde hydrogenation over the Pd_1_/TiO_2_ catalyst. Moreover, the tunneling probability was found to be regulatable by adjusting the tunneling width and barrier height through the use of different protic solvents and to be enhanced by exciting hydrogen bonds to higher vibrational energy levels via FIR irradiation.

## Methods

### Preparation of Ultrathin TiO_2_(B) Nanosheets

TiO_2_(B) nanosheets were prepared according to the method by Zheng and co-workers.^43^ 1 mL of TiCl_4_ was added to 30 mL of ethylene glycol under stirring. After stirring for 10 min, 1 mL of water was then introduced into the mixture. After stirring for another 10 min, the solution was transferred to a 20 mL Teflon-lined stainless-steel autoclave. The sealed vessel was then heated from room temperature to 423 K and kept for 2 h before it was cooled to room temperature. The white products were collected via centrifugation and further washed with water and ethanol. After being dried in a vacuum oven at 333 K, the TiO_2_(B) nanosheets were used for further preparation.

### Preparation of Pd_1_/TiO_2_ Catalyst

A H_2_PdCl_4_ aqueous solution (1 mol/L) was prepared by reacting PdCl_2_ with concentrated HCl stoichiometrically at 70 °C for 1 h, which was then diluted to 0.005 mol/L.^60^ The Pd_1_/TiO_2_ catalysts were prepared according to the method by Zheng and co-workers.^43^ TiO_2_(B) nanosheets (15 mg) were dispersed in deionized water (20 mL). A H_2_PdCl_4_ solution (100 μL, 0.005 M, 0.5 μmol of Pd) was added to the dispersion under vigorous stirring. After aging for 15 min, the mixture was then treated by the 365 nm UV light. After 10 min of irradiation, the light gray product was collected via centrifugation and further washed by water for 3 times. After being dried in vacuum oven at 333 K for 6 h, the obtained sample was sealed and stored in a 10 mL centrifuge tube for further characterization and application. Of note, the UV treatments were carried out on a Xenon-lamp parallel light source system equipped with an optical filter allowing 365 nm light transmission.

### Catalytic Reaction

In a typical catalytic experiment, a 48 mL glass pressure vessel equipped with a stirrer (IKA C-MAG HS 7) was employed as a reactor. In a typical reaction, 3.0 mg of Pd_1_/TiO_2_ (0.1 μmol Pd) was dispersed into 5 mL of CH_3_OH, and 0.1 MPa H_2_ (controlled by the pressure reducing valve) was charged at 303 K for 15 min. 10.2 μL (100 μmol) of benzaldehyde was then added. After a given time, aliquots of 100 μL were periodically taken and centrifuged for further analysis by gas chromatography (GC), high performance liquid chromatography (HPLC), or ^1^H NMR. The conversion and selectivity could be easily and accurately obtained by integrating peak areas in these methods. For the different solvents (i.e, water, *tert*-butanol, cyclohexanol, and tetrahydrofuran), 5 mL of CH_3_OH was replaced while the other reaction conditions were kept the same. For the different gases (H_2_ and D_2_), the pressure was calibrated by a digital pressure gauge (HG-808XB) to 0.100 MPa. For the hydrogenation of other substrates, the reaction conditions were the same as for benzaldehyde hydrogenation while 100 μmol of different substrates was used. For the catalyst recycling test, after the first cycle, the same amount of benzaldehyde was added to the reactor to carry out the second run. The recycling test was repeated for another four runs. To apply FIR irradiation to the hydrogenation of benzaldehyde, the heating film of graphene was electrically powered (5 V) at 1, 3, or 5 W. The reaction temperature was kept at 273 K by circulating cooling water to keep the temperature fluctuation at ±0.05 °C.

### Computational Details

DFT calculations were performed by using the Gaussian 16 program.^61^ The ground and transition state geometrical structures were optimized by hybrid exchange correlation functional B3LYP. While the 6-31G* basis set was adopted for H, C, and O atoms, the LANL2DZ basis set was adopted for Pd atoms. The solvation model of density (SMD) was adopted for all calculations in solvent CH_3_OH and H_2_O with a dielectric constant of 32.613 and 78.355, respectively. The vibration frequency calculation results show that all of the ground state structures had no imaginary frequency. The transition state structures had only one imaginary frequency. The structures of the transition state were verified by intrinsic reaction coordinate analysis. The reaction rate constants were calculated by eq 1.

1 $$k=\Gamma \frac{k_{B}T}{h}e^{-\Delta G/RT}$$

where *k*, *k*_B_, *T*, *h*, Γ, and Δ*G* is reaction rate constant, Boltzmann constant, Kelvin temperature, Planck constant, quantum tunneling correction factor, and reaction Gibbs free energy of activation, respectively. The energy barrier has been corrected by considering zero-point energies in the calculation.

The quantum tunneling correction factor was calculated as the Eckart potential function;^62^ the factor is defined as the ratio between quantum mechanical rate and classical mechanical rate and can be written as eq 2:

2 $$\Gamma =e^{-\Delta V_{1}/k_{B}T}\int_{0}^{\infty }e^{-E/k_{B}T}\kappa \left(E\right)\;d\left(E/k_{B}T\right)$$

where Δ*V*_1_ is the energy barrier from transition state to the reaction initial state and κ­(*E*) is the transmission probability (eq 3),

3 $$\kappa \left(E\right)=1-\frac{\cosh2\pi \left(a-b\right)+\cosh2\pi d}{\cosh2\pi \left(a+b\right)+\cosh2\pi d}$$

where

4 $$\begin{array}{l} 2\pi a=\frac{2\sqrt{\alpha_{1}\xi }}{\sqrt{\alpha_{1}}+\sqrt{\alpha_{2}}}\\ 2\pi b=\frac{2\sqrt{\left(1+\xi \right)\alpha_{1}-\alpha_{2}}}{\sqrt{\alpha_{1}}+\sqrt{\alpha_{2}}}\\ 2\pi d=2\sqrt{\alpha_{1}\alpha_{2}-4\pi^{2}/16}\\ \xi =E/\Delta V_{1} \end{array}$$

In eq 4,

5 $$\begin{array}{l} \alpha_{1}=2\pi \Delta V_{1}/hv^{*}\\ \alpha_{1}=2\pi \Delta V_{2}/hv^{*}\\ v^{*}=\left(1/2\pi \right){\left(-F/m\right)}^{1/2}\\ -F=\frac{\pi^{2}\left[{\left(\Delta V_{1}-\Delta V_{2}\right)}^{2}-{\left(\sqrt{\Delta V_{1}}+\sqrt{\Delta V_{2}}\right)}^{4}\right]}{2L^{2}{\left(\sqrt{\Delta V_{1}}+\sqrt{\Delta V_{2}}\right)}^{4}} \end{array}$$

In eq 5, *F* is the second derivative of the potential energy function evaluated at its maximum and Δ*V*_1_ and Δ*V*_2_ are the energy barriers from the transition state to the reaction initial state and from the transition state to the reaction final state, respectively.

To verify the reliability of the B3LYP method for this system, calculations were performed using the CAM-B3LYP, M06, and B3LYP functions. The calculated KIE (*k*_H2, CH3OH_/*k*_D2, and CD3OD_) values are shown in Table S7. The results show that the B3LYP function is more accurate for this system because the KIE (*k*_H2, CH3OH_/*k*_D2, CD3OD_) value (60) calculated by B3LYP is in better agreement with the experimental results.

The PIMD simulations were performed using an artificial intelligence-enhanced molecular simulation framework with the graph field network (GFN) force field.^63^ The detailed neural network parameters for the force field are provided in Table S8. A dataset of 4,669 configurations was employed to train the final force field. All DFT calculations for the conformations in the dataset were performed at the wB97M-V/def2-TZVP level using ORCA 6.0.^64–67^ The force root-mean-square error (RMSE) across the whole dataset is 1.47 kcal/mol/Å (Figure S25). All PIMD simulations were carried out using the MindSPONGE simulation package.^63^ The simulations were conducted at 300 K using 32 beads to ensure an accurate representation of the nuclear quantum effect. Temperature control was implemented using the PILE thermostat with a time constant set to 0.1, and the time step was set to 0.5 fs. Metadynamics was used to accelerate sampling of the reaction. We employed a bias potential updated every 0.1 ps with a height of 2 kJ/mol and a bias factor of 80. The collective variable selected to capture the reaction in the metadynamics simulations was defined as eq 6.

6 $$CV=d_{O^{\left(3\right)}H^{\left(2\right)}}+d_{O^{\left(2\right)}H^{\left(1\right)}}-d_{O^{\left(1\right)}H^{\left(1\right)}}-d_{O^{\left(2\right)}H^{\left(2\right)}}$$

where the superscripted numbers correspond to the atom indices labeled in Figure S26. We employ a practical version of QTST where the rate constant is calculated by eq 7.

7 $$k_{QTST}=\frac{\mathrm{\nu ̅}}{2}P\left(\xi^{\#},T\right)=\frac{\mathrm{\nu ̅}}{2}P\left(\xi^{0},T\right)e^{-\Delta F/k_{B}T}$$

where the thermal velocity $\mathrm{\nu ̅}=\sqrt{2k_{B}T/\pi m}$ . *P*(ξ^#^,*T*) and *P*(ξ^0^,*T*) refers to the centroid probability densities at the transition state ξ^#^ and initial state ξ^0^, respectively, and Δ*F* is the free energy barrier for the proton transfer.

The KIE values are calculated using eq 8:

8 $$\frac{k_{H}}{k_{D}}=\sqrt{\frac{m_{D}}{m_{H}}}\frac{P{\left(\xi^{0},T\right)}_{H}}{P{\left(\xi^{0},T\right)}_{D}}e^{-\left[(\Delta F_{H}-\Delta F_{D}\right)/k_{B}T]}$$

In Figures S9–S13, to construct the 2D free energy profile, we introduced two additional CVs (eqs 9 and 10) to analyze the hydrogen transfer processes:

9 $$CV_{1}=d_{O^{\left(3\right)}H^{\left(2\right)}}-d_{O^{\left(2\right)}H^{\left(2\right)}}$$

representing hydrogen transfer between the solvent molecule and product and

10 $$CV_{2}=d_{O^{\left(2\right)}H^{\left(1\right)}}-d_{O^{\left(1\right)}H^{\left(1\right)}}$$

representing transfer between the catalyst and solvent molecule. The superscripted numbers correspond to the atom indices labeled in Figure S26.

## Supplementary Material



## Notes

Materials, characterizations, figures (S1–S26), and tables (S1–S9) for experimental and computational results and atomic coordinates of computational models (PDF)
