# Supplementary material for: Catalytic Hydrogenation Dominated by Concerted Hydrogen Tunneling at Room Temperature
Source: ACS Cent Sci. 2025 Sep 26;11(11):2180–7. doi: 10.1021/acscentsci.5c00943 (PMC12670312; doi:10.1021/acscentsci.5c00943)
Supplement: Supplementary file 1 [file oc5c00943_si_001.pdf]

Supporting Information for

Catalytic Hydrogenation Dominated by Concerted Hydrogen Tunneling at Room Temperature

Qingyuan Wu,[1†] Pengxin Liu,[2†] Xia-Guang Zhang,[3†] Cheng Fan,[4†] Ziwen Chen,[1] Ruixuan Qin,[1,5] Yi Qin Gao,[4,6*] Yi Zhao,[1*] Nanfeng Zheng[1,5*]

Affiliations:

[1] New Cornerstone Science Laboratory, State Key Laboratory for Physical Chemistry of Solid Surfaces, iChEM, and National & Local Joint Engineering Research Center for Preparation Technology of Nanomaterials, College of Chemistry and Chemical Engineering, Xiamen University, Xiamen 361005, China.

[2] School of Physical Science and Technology, ShanghaiTech University, Shanghai 201210, China.

[3] Key Laboratory of Green Chemical Media and Reactions, Ministry of Education, Collaborative Innovation Center of He-nan Province for Green Manufacturing of Fine Chemicals, School of Chemistry and Chemical Engineering, Henan Normal University, Xinxiang 453007, China.

[4] Institute of Theoretical and Computational Chemistry, College of Chemistry and Molecular Engineering, Peking University, Beijing 100871, China.

[5] Innovation Laboratory for Sciences and Technologies of Energy Materials of Fujian Province (IKKEM), Xiamen 361102, China.

[6] Changping Laboratory, Beijing 102200, China.

[†] These authors contributed equally to this work.

[*] Corresponding authors. Emails: nfzheng@xmu.edu.cn (N. F. Zheng); yizhao@xmu.edu.cn (Y. Zhao); gaoyq@pku.edu.cn (Y. Q. Gao).

Materials

Titanium tetrachloride ($TiCl_4$, A. R.), ethylene glycol ($C_2H_6O_2$, A. R.), palladium chloride [$PdCl_2$, 59.8%] and Pd/C were purchased from J&K Scientific Ltd. (Bejing, China). Deuterium oxide (D_2O, A. R.), methanol (CH_4O, A. R.), methanol-D4 (CD_4O, A. R.), tert-butanol ($C_4H_{10}O$, A. R.), cyclohexanol ($C_6H_{12}O$, A. R.) and anhydrous tetrahydrofuran (C_4H_8O, A. R.) were purchased from Sinopharm Chemical Reagent Co. Ltd. (Shanghai, China). Benzaldehyde (C_7H_6O, A. R.), p-methylbenzaldehyde (C_8H_8O, A. R.), 4-hydroxybenzaldehyde ($C_7H_6O_2$, A. R.), 4-methoxybenzaldehyde ($C_8H_8O_2$, A. R.), 4-(acetyloxy)benzaldehyde ($C_9H_8O_3$, A. R.), 4-fluorobenzaldehyde (C_7H_5FO, A. R.), 3-hydroxybenzaldehyde ($C_7H_6O_2$, A. R.), and 3-methoxybenzaldehyde ($C_8H_8O_2$, A. R.) was purchased from Alfa Aesar Chemical Reagent Co. Ltd. (Tianjin, China). H_2 (99.999%) and D_2 (99.999%) were purchased from Linde Gas. The water used in all experiments was ultrapure (18.25 MΩ). All reagents were used without further purification. The graphene films (XHGHF-C3) used in this work were obtained from Xiamen XiHe Technology Co., Ltd.

HPLC analysis: The reaction was stopped after desired time to extract 10 μL solutions for liquid chromatographic analysis. C18 column, mobile phase: 50% water and 50% acetonitrile, column temperature: 30°C, UV wavelength: 210 nm.

^{1}H NMR spectra characterizations: ^{1}H NMR spectra was recorded on an AVANCE III 500 MHz spectrometer. All NMR data were processed on MestReNova software.

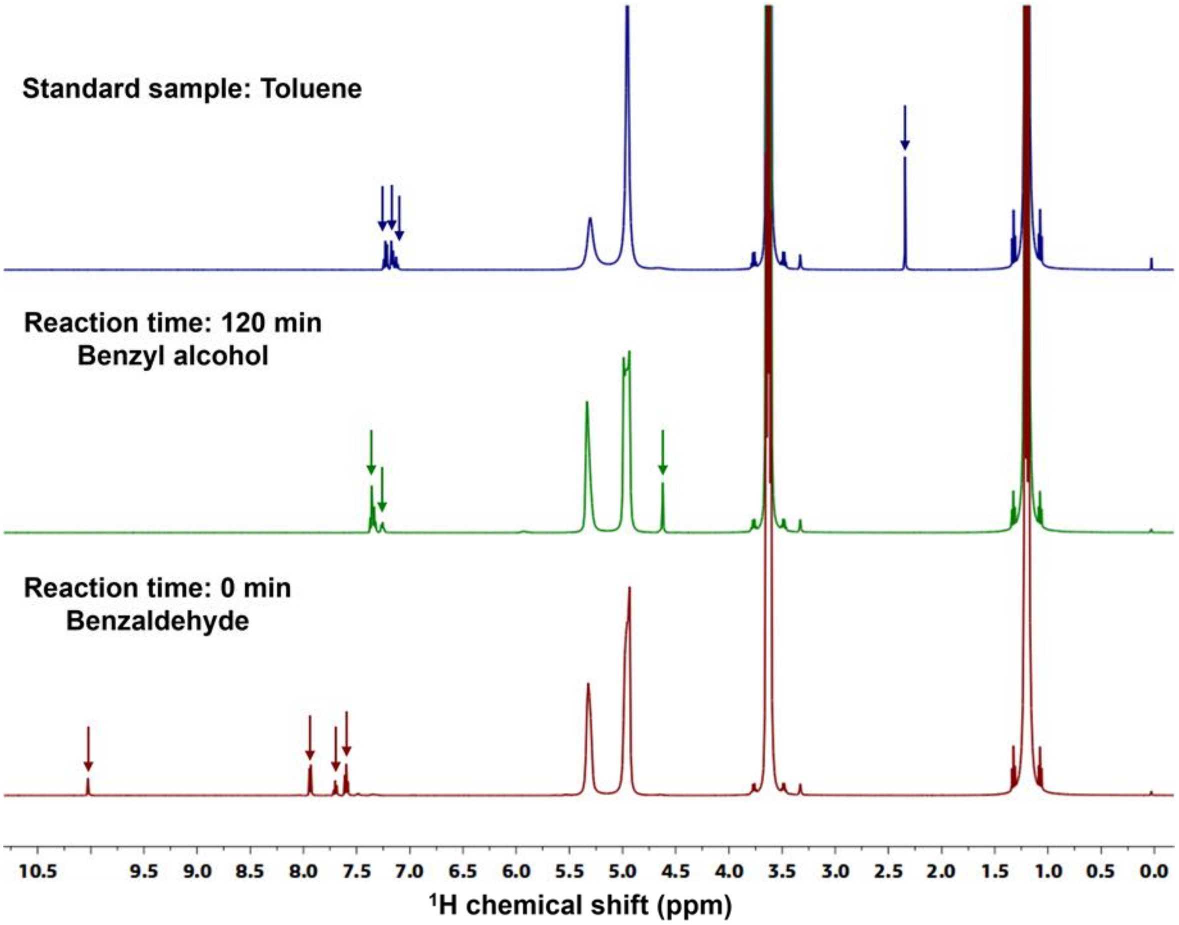

Figure S1. Monitoring of benzaldehyde hydrogenation by ^{1}H-NMR in CD_3OD. Brown, green and blue arrows indicate benzaldehyde, benzyl alcohol and toluene (standard sample), respectively. The peaks without arrows are from the solvents. Benzaldehyde was fully converted to benzyl alcohol within 60 min. Even after extending the reaction time to 120 min, no over-hydrogenated toluene was detected, as evidenced by the absence of its characteristic methyl signal at 2.68 ppm in the ^{1}H NMR spectrum.

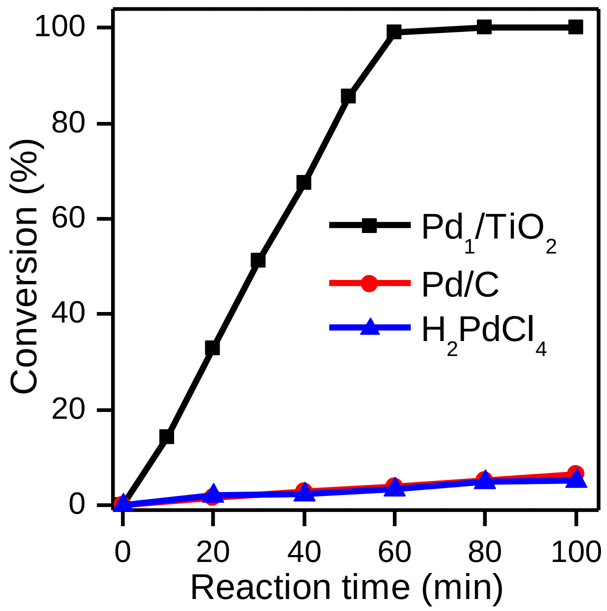

Figure S2. Catalytic performances of Pd_1/TiO_2, Pd/C and H_2PdCl_4 in benzaldehyde hydrogenation. While Pd_1/TiO_2 readily converted all benzaldehyde into benzyl alcohol in 60 min, both Pd/C and H_2PdCl_4 showed much lower activities under the same conditions. Reaction conditions: 5 mL CH_3OH, 0.1 μmol Pd, 100 μmol benzaldehyde, 303 K, 0.1 MPa H_2.

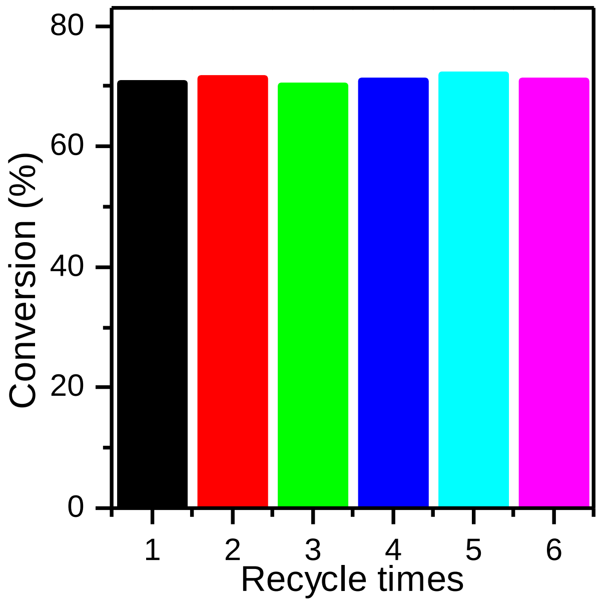

Figure S3. The recycling test of Pd_1/TiO_2 for benzaldehyde hydrogenation. Reaction time: 40 min. No activity decrease was observed in the six recycling tests. After the first cycle, the same amount of benzaldehyde was added to the reactor to carry out the second run. The recycling test was repeated for another four runs.

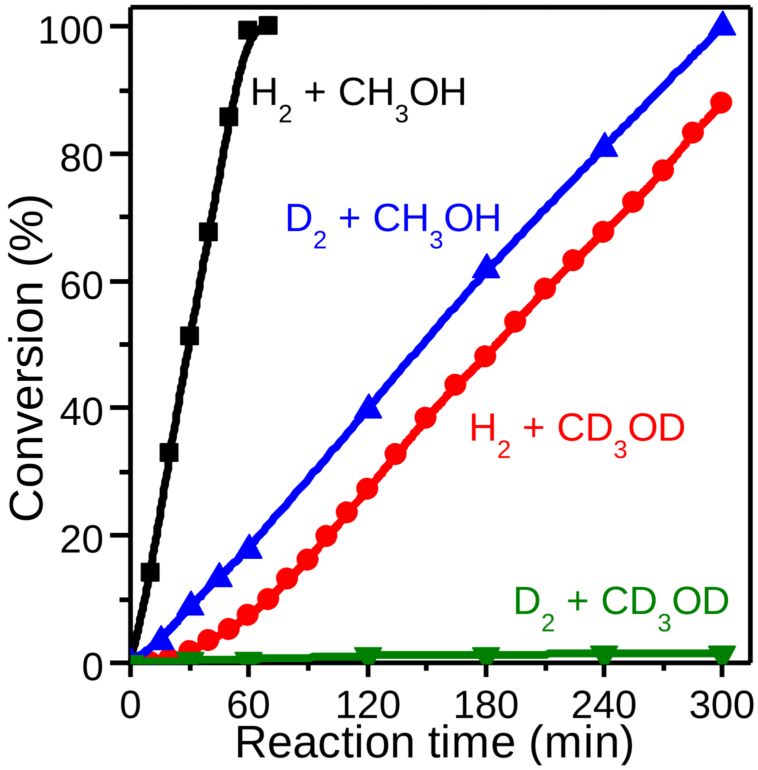

Figure S4. The conversion of benzaldehyde catalyzed by Pd_1/TiO_2 via using (H_2, CH_3OH), (D_2, CH_3OH), (H_2, CD_3OD) and (D_2, CD_3OD) at 303 K. An induction period was observed when H_2 and CD_3OD were used during 0-60 min (red curve). PIMD simulations indicate that the tunneling of solvent protons to reactant is the more dominant process in the concerted triple H-tunneling, so the initial kinetic impediment is attributed to the inefficient transfer of deuterium from CD_3OD to the reactant. However, hydrogen-deuterium exchange led to the conversion of CD_3OD to CD_3OH, resulting in a gradual acceleration of the reaction rate until the rate was similar to that of the system using D_2 and CH_3OH. The benzaldehyde was completely converted to benzyl alcohol in ~60 min in CH_3OH at 303 K under H_2 (black curve). However, the conversion of benzaldehyde was minimal even after 300 min, when both H_2 and CH_3OH were replaced by D_2 and CD_3OD, respectively (green curve).

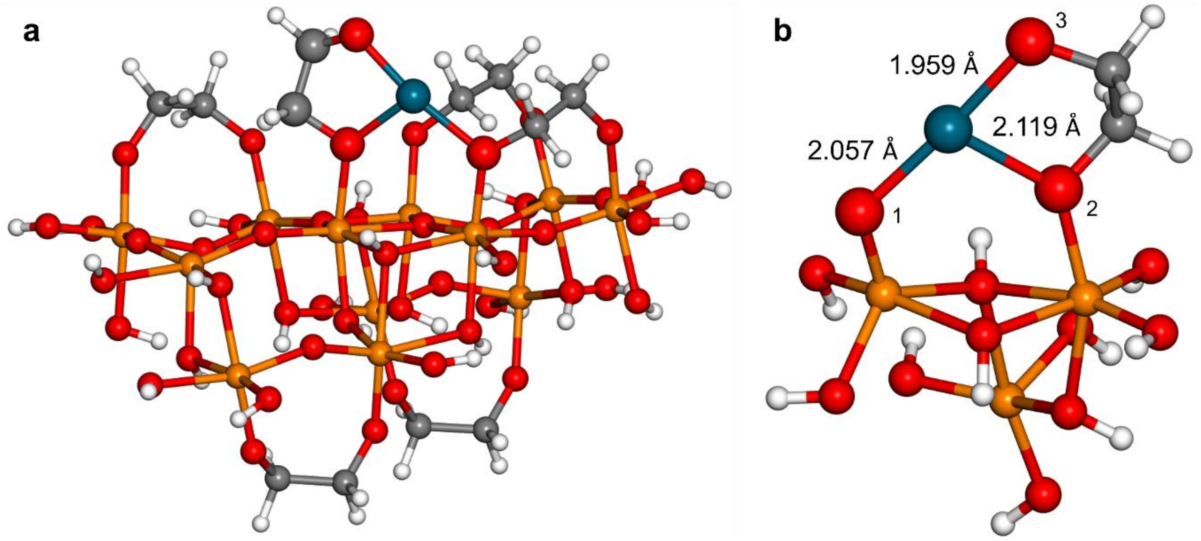

Figure S5. (a) The cluster model taken from the periodic structure (Science 2016, 352, 797-801). (b) The simplified cluster model of the catalyst active site. Turquoise, red, yellow, grey and white balls indicate Pd, O, Ti, C and H atoms, respectively. The cluster model (a) consists of two TiO_2 layers with EG adsorbed on the surface. The outer edge oxygen atoms are saturated with hydrogen atoms. The Pd atom is adsorbed on the oxygen atom of EG and two oxygen atoms of another EG. The bond lengths of Pd-O are 2.067, 2.108 and 1.907 Å respectively. Similarly, the Pd atom in the optimized simplified model (b) is coordinated with three oxygen atoms with the Pd-O bond lengths of 2.057 (O1), 2.119 (O2) and 1.959 (O3) Å, respectively. It is thus to adopt the simplified model (b) for reducing the computation cost.

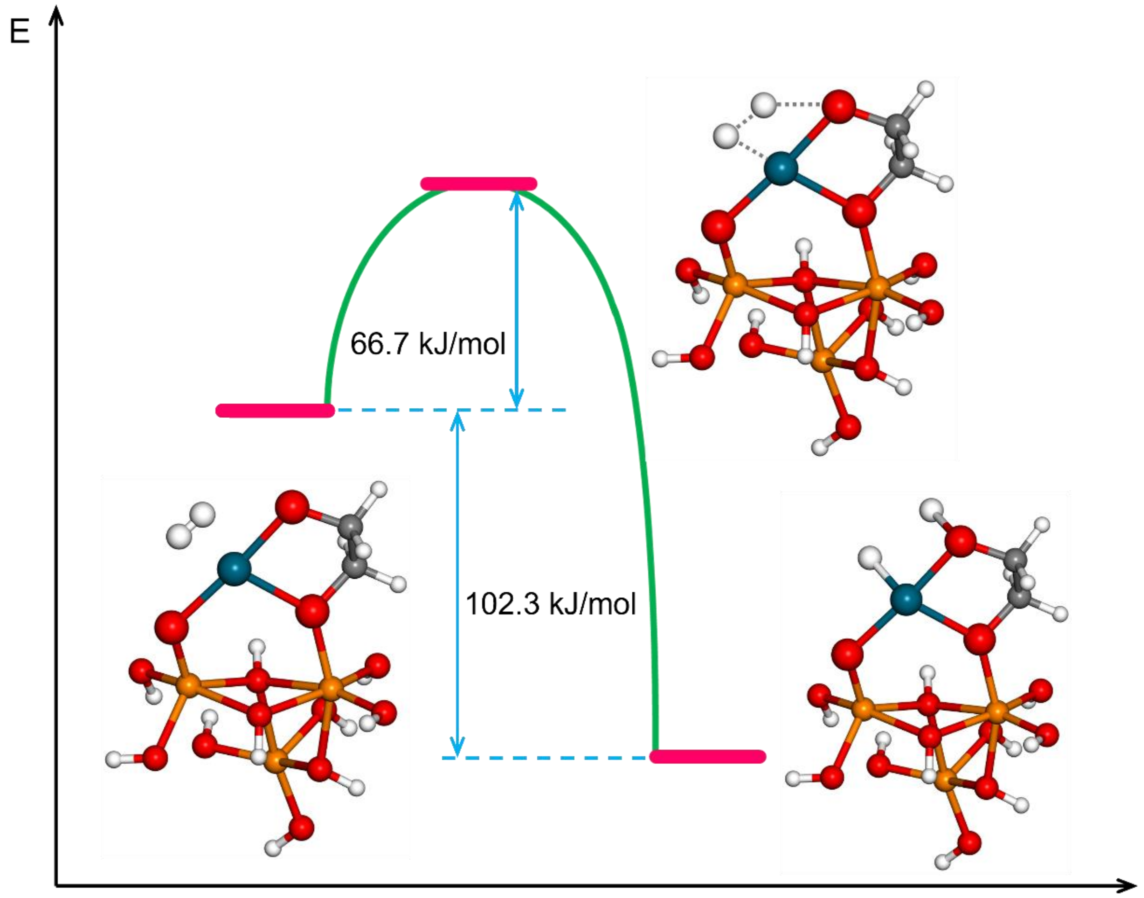

Figure S6. Energy profile for the heterolytic H_2 activation process for Pd_1/TiO_2 and structures of different states. Turquoise, red, yellow, grey and white balls indicate Pd, O, Ti, C and H atoms, respectively. The bond lengths of Pd-H, H-H, O-H of transition state are 1.750, 0.991 and 1.378 Å, respectively. The imaginary frequency of transition state is 1570 cm^{-1}. The activation of H_2 on the simplified model is an exothermic reaction with an energy value of ~102.3 kJ/mol and an energy barrier of ~66.7 kJ/mol. Once H_2 activated, two hydrogen atoms are adsorbed at Pd-O interface with one on the Pd atom and the other on the O atom of EG. The natural population analysis reveals that one hydrogen atom on Pd is $H^{\delta-}$ while the other is $H^{\delta+}$.

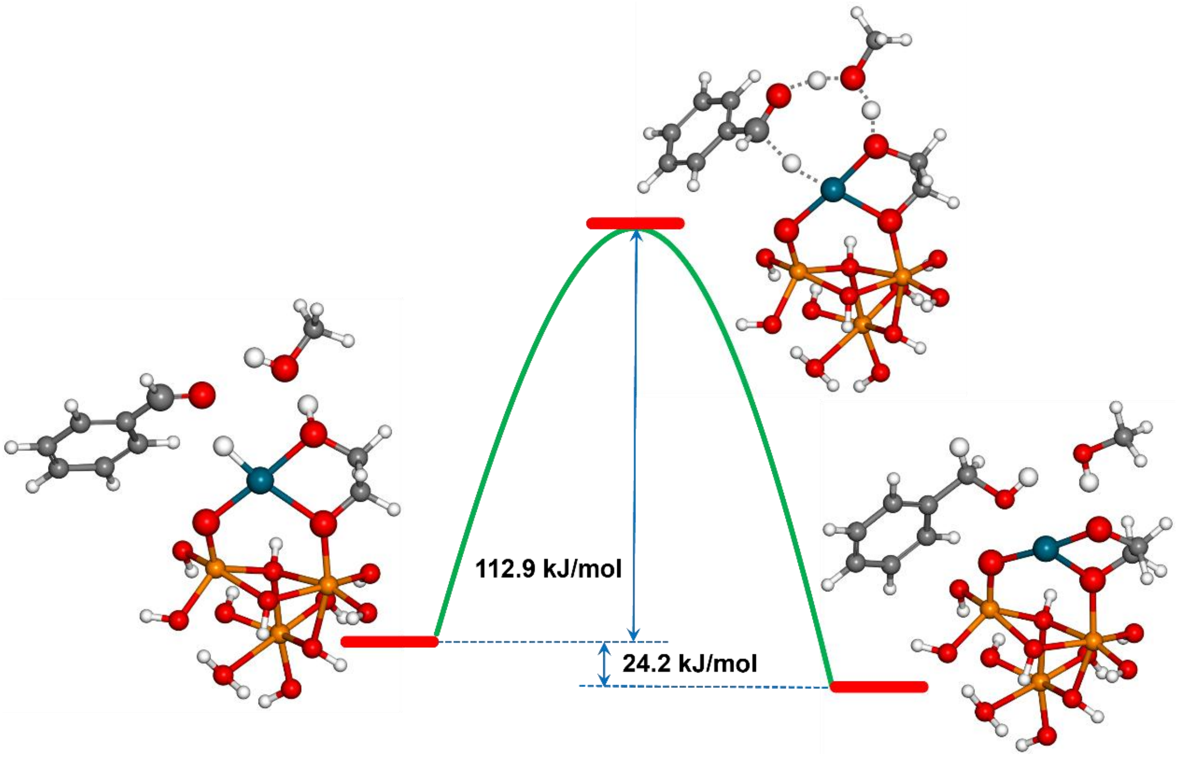

Figure S7. Energy profile for hydrogenation via the H-tunneling mechanism starting from heterolytic dissociation of H_2 on Pd_1/TiO_2 in CH_3OH. Turquoise, red, yellow, grey and white balls indicate Pd, O, Ti, C and H atoms, respectively. The reaction barrier was calculated to be ~112.9 kJ/mol. The reaction was found to be a slightly exothermic process with an overall energy of ~24.2 kJ/mol. The imaginary frequency of transition state was calculated to be 1306 cm^{-1}.

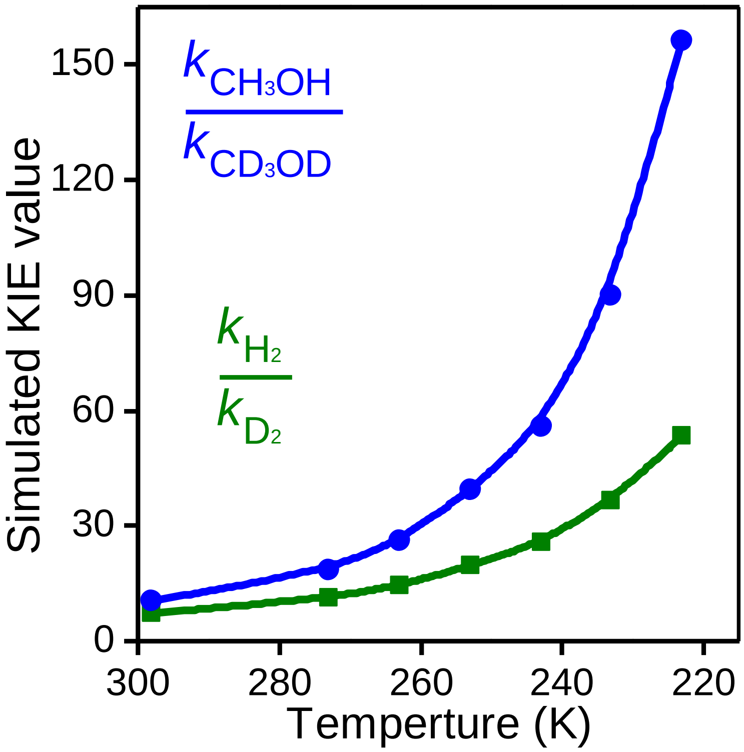

Figure S8. Simulated KIE (k_{H2}/k_{D2}) and KIE (k_{CH3OH}/k_{CD3OD}) values at different temperatures. The KIE (k_{H2}/k_{D2}) values at 298, 273, 263, 253, 243, 233 and 223 K were simulated to be 7.0, 11.1, 14.3, 19.5, 25.8, 36.3 and 53.6, respectively. The KIE (k_{CH3OH}/k_{CD3OD}) values at 298, 273, 263, 253, 243, 233 and 223 K were simulated to be 10.5, 18.5, 26.1, 39.3, 55.9, 89.9 and 156, respectively. The calculated absolute rate constants were summarized in Table S4.

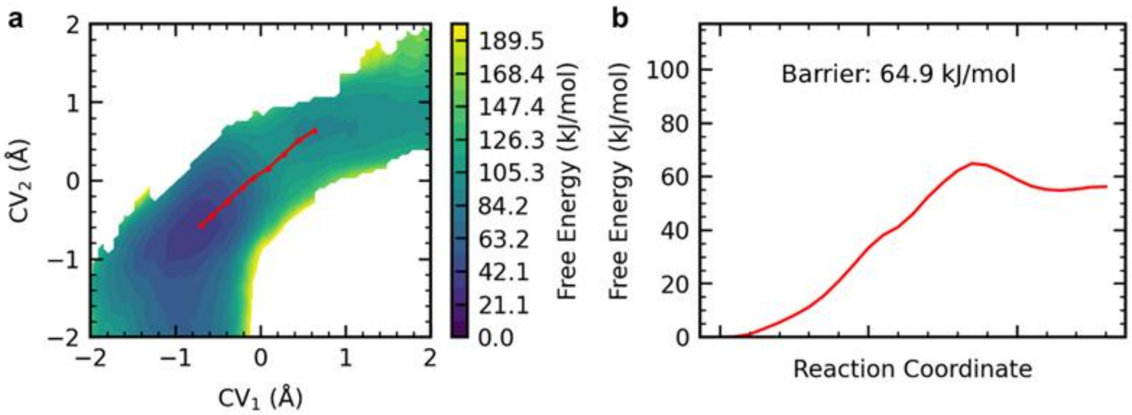

Figure S9. (a) PIMD simulation of 2D free energy surfaces (FES) when CH_3OH and H_2 were used. The red line traces the minimum energy path (MEP), representing the most favorable reaction pathway, while the contour spacing reflects energy barriers, with lighter regions indicating higher-energy transition states or intermediates. (b) The corresponding 1D free energy profile along the MEP, quantifying the thermodynamic changes (ΔG) during the reaction. The energy barrier for the reaction was determined to be 64.9 kJ/mol.

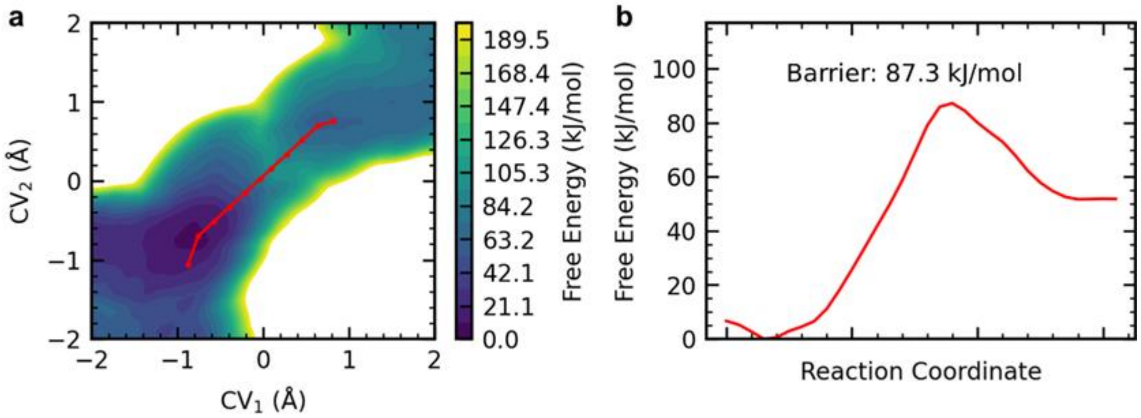

Figure S10. (a) Classical MD simulation of 2D FES when CH_3OH and H_2 were used. The red line traces the MEP, representing the most favorable reaction pathway, while the contour spacing reflects energy barriers, with lighter regions indicating higher-energy transition states or intermediates. (b) The corresponding 1D free energy profile along the MEP, quantifying the ΔG during the reaction. The energy barrier for the reaction was determined to 87.3 kJ/mol.

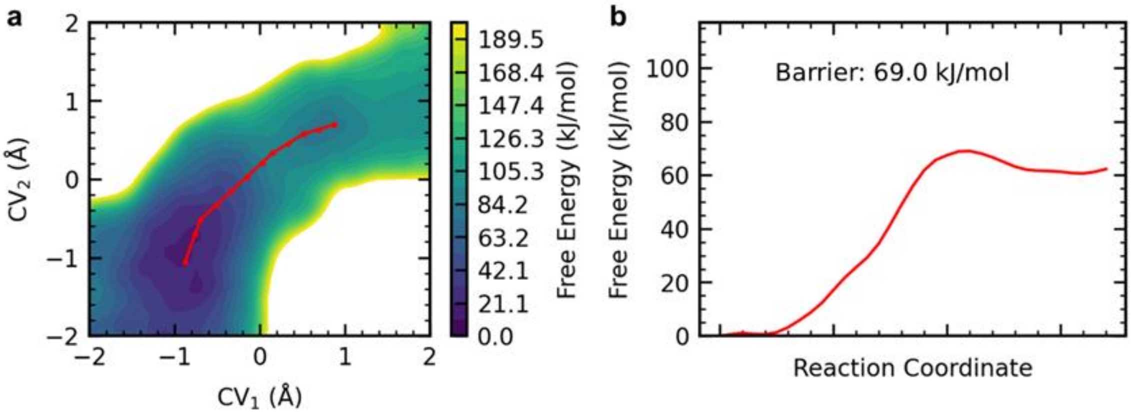

Figure S11. (a) PIMD simulation of 2D free energy surfaces (FES) when CH_3OH and D_2 were used. The red line traces the MEP, representing the most favorable reaction pathway, while the contour spacing reflects energy barriers, with lighter regions indicating higher-energy transition states or intermediates. (b) The corresponding 1D free energy profile along the MEP, quantifying the ΔG during the reaction. The energy barrier for the reaction was determined to be 69.0 kJ/mol.

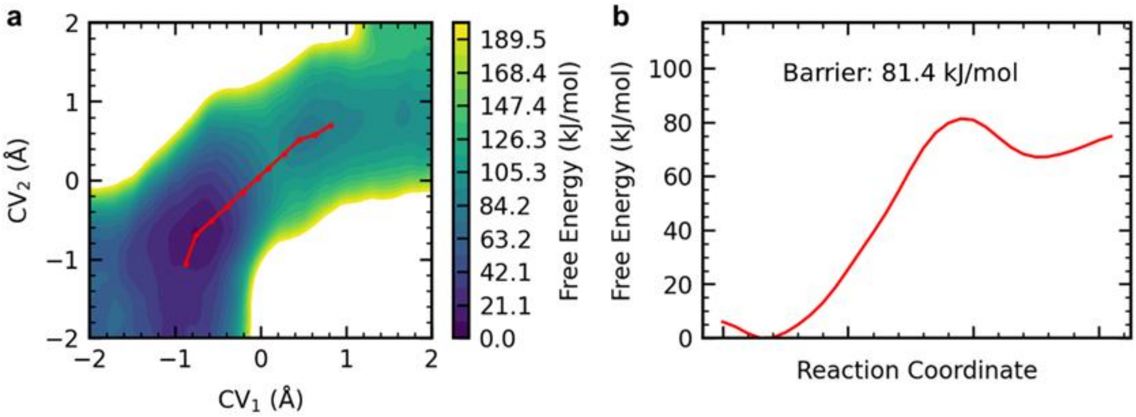

Figure S12. (a) PIMD simulation of 2D free energy surfaces (FES) when CD_3OD and H_2 were used. The red line traces the MEP, representing the most favorable reaction pathway, while the contour spacing reflects energy barriers, with lighter regions indicating higher-energy transition states or intermediates. (b) The corresponding 1D free energy profile along the MEP, quantifying the ΔG during the reaction. The energy barrier for the reaction was determined to be 81.4 kJ/mol.

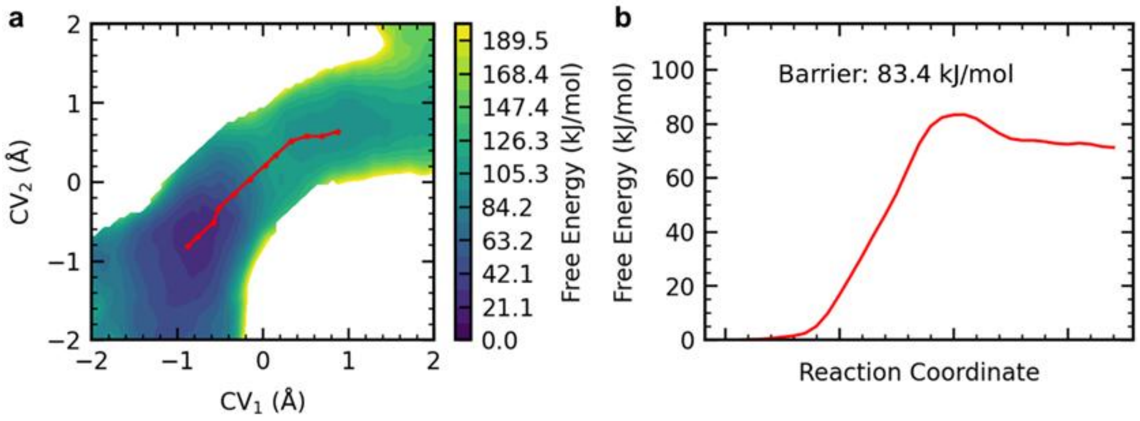

Figure S13. (a) PIMD simulation of 2D free energy surfaces (FES) when CD_3OD and D_2 were used. The red line traces the MEP, representing the most favorable reaction pathway, while the contour spacing reflects energy barriers, with lighter regions indicating higher-energy transition states or intermediates. (b) The corresponding 1D free energy profile along the MEP, quantifying the ΔG during the reaction. The energy barrier for the reaction was determined to 83.4 kJ/mol.

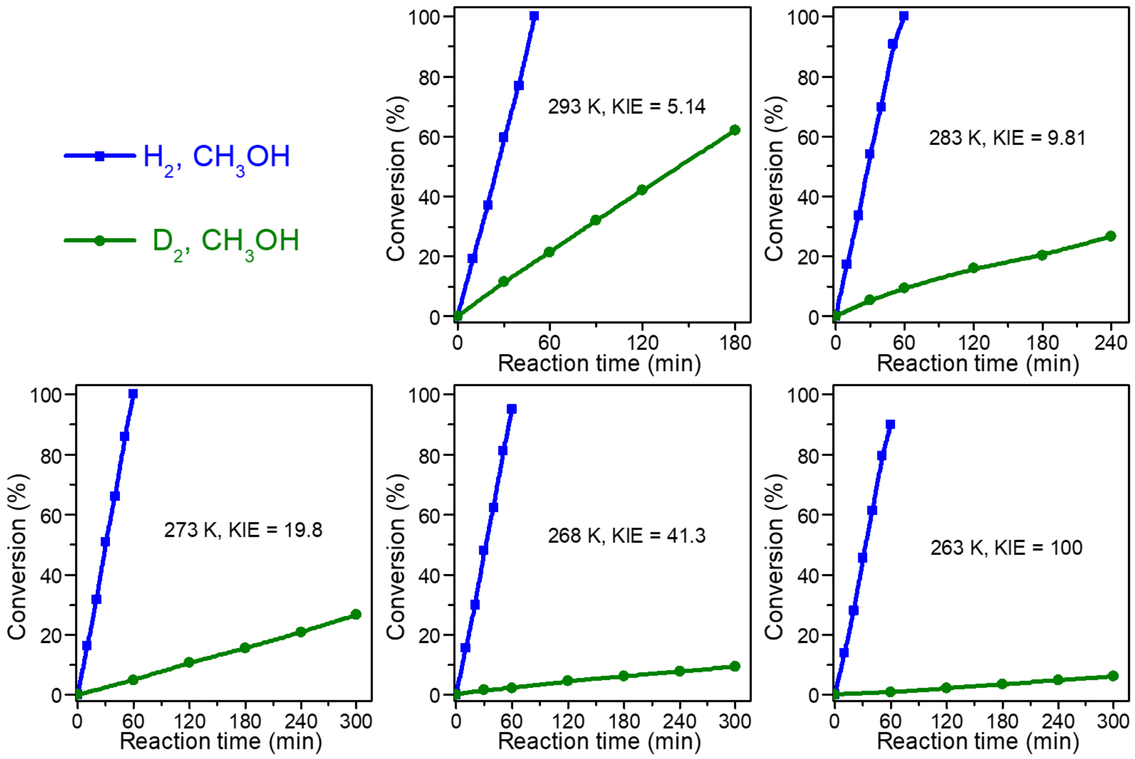

Figure S14. Experimentally measured KIE (k_{H2}/k_{D2}) values at different temperatures. The KIE (k_{H2}/k_{D2}) values at 293, 283, 273, 268 and 263 K were 5.14, 9.81, 19.8, 41.3 and 100, respectively. Reaction conditions: 5 mL CH_3OH, 0.1 μmol Pd, 100 μmol benzaldehyde, 0.1 MPa H_2 or D_2.

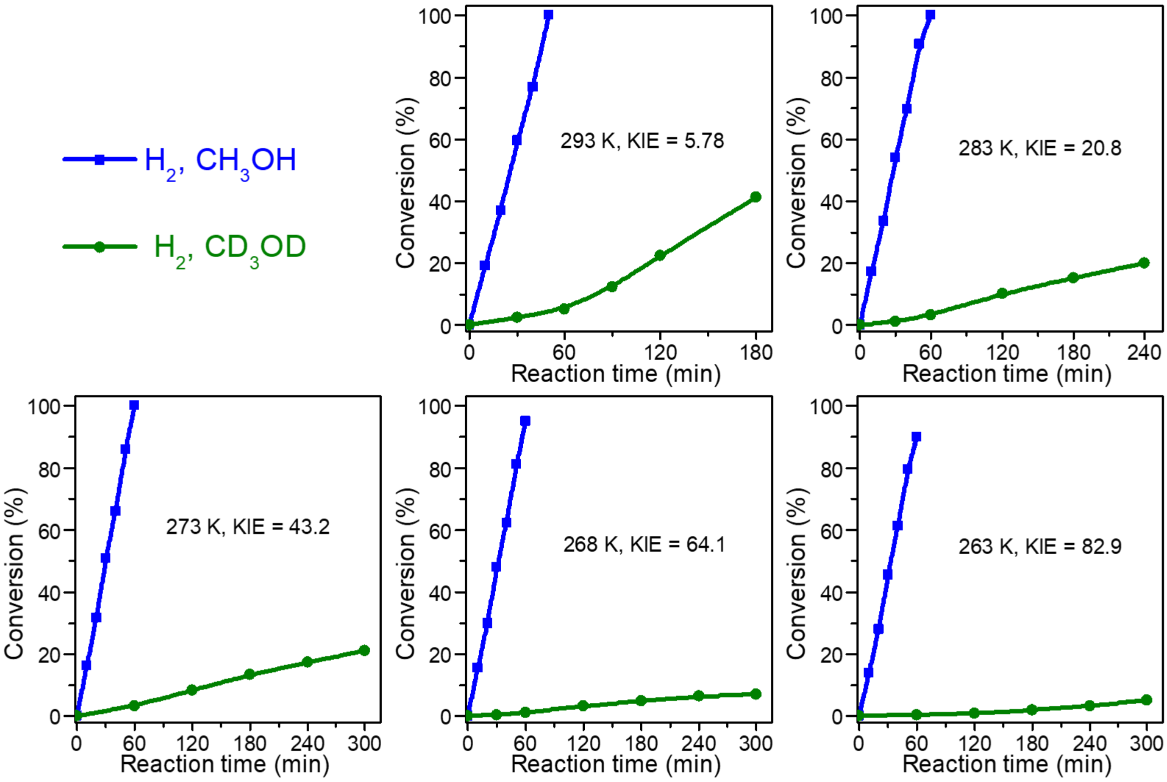

Figure S15. Experimentally measured KIE (k_{CH3OH}/k_{CD3OD}) values at different temperatures. The KIE (k_{CH3OH}/k_{CD3OD}) values at 293, 283, 273, 268 and 263 K were 5.78, 20.8, 43.2, 64.1 and 82.9, respectively. Reaction conditions: 5 mL CH_3OH or CD_3OD, 0.1 µmol Pd, 100 µmol benzaldehyde, 0.1 MPa H_2.

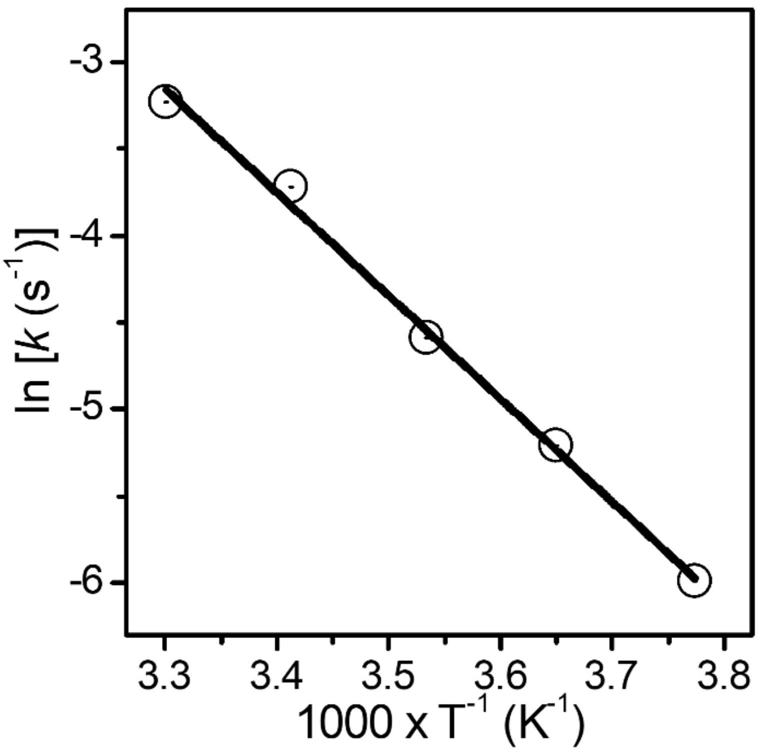

Figure S16. The Arrhenius plot of benzaldehyde hydrogenation over the Pd/C catalyst by using H_2 and CH_3OH. No detectable slope change was observed. Reaction conditions: 5 mL CH_3OH, 0.1 μmol Pd, 100 μmol benzaldehyde, 0.1 MPa H_2. The R^2 of linear fitting is 0.995.

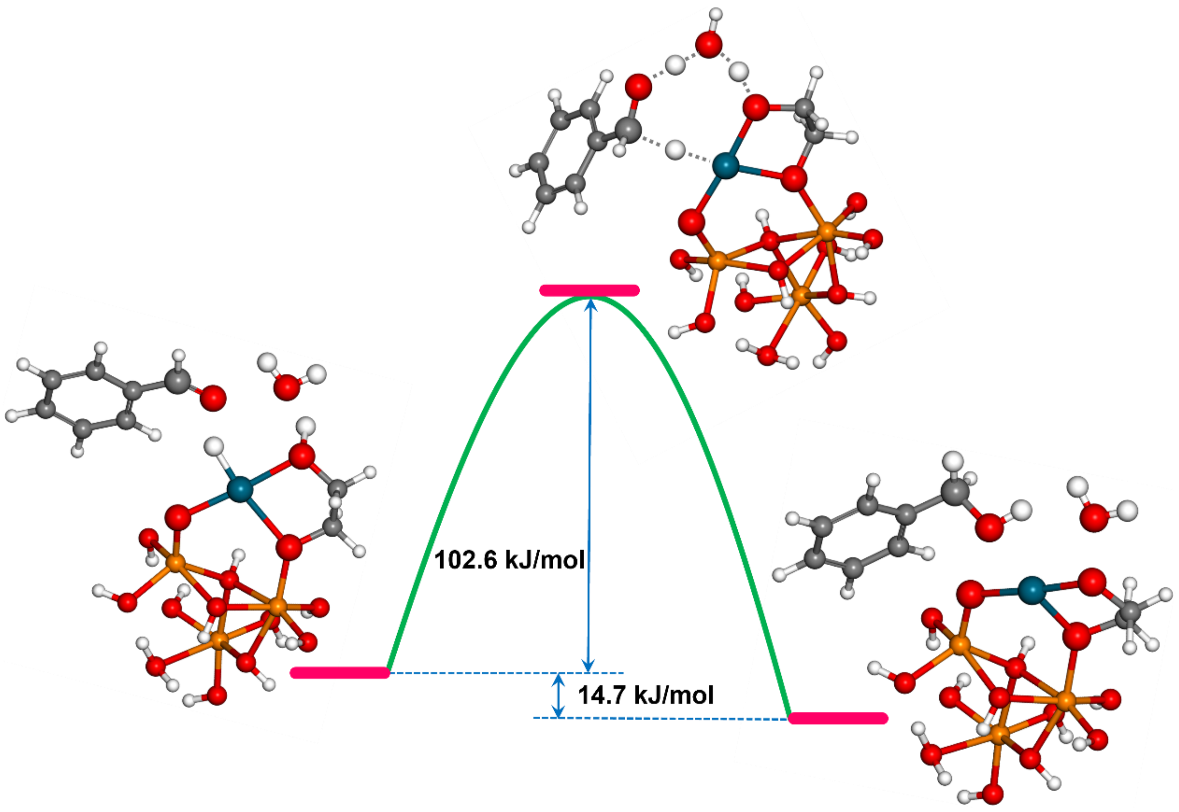

Figure S17. Energy profile for the benzaldehyde hydrogenation via the H-tunneling mechanism starting from heterolytic dissociation of H_2 on Pd_1/TiO_2 in H_2O. Turquoise, red, yellow, grey and white balls indicate Pd, O, Ti, C and H atoms, respectively. The reaction barrier was determined to be ~102.6 kJ/mol. The reaction is found to be a slightly exothermic process with an overall energy of ~14.7 kJ/mol. The imaginary frequency of transition state was calculated to be 1335 cm^{-1}.

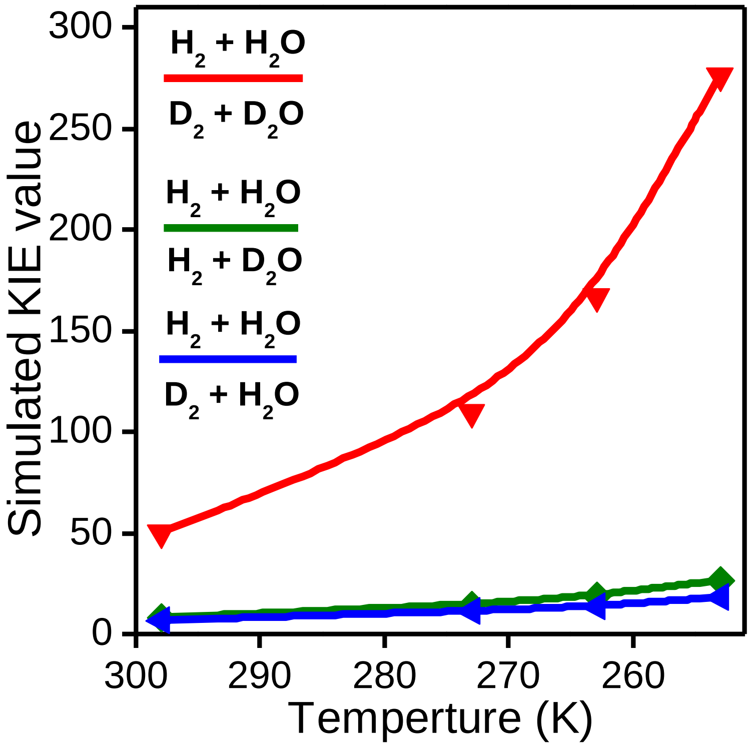

Figure S18. Simulated KIE ($k_{H2, H2O}/k_{D2, H2O}$), KIE ($k_{H2, H2O}/k_{H2, D2O}$) and KIE ($k_{H2, H2O}/k_{D2, D2O}$) values at different temperatures. The KIE ($k_{H2, H2O}/k_{D2, H2O}$) values at 298, 273, 263 and 253 K were simulated to be 7.2, 11.1, 14.0 and 18.4, respectively. The KIE ($k_{H2, H2O}/k_{H2, D2O}$) values at 298, 273, 263 and 253 K were simulated to be 8.6, 14.7, 19.4 and 27.0, respectively. The KIE ($k_{H2, H2O}/k_{D2, D2O}$) values at 298, 273, 263 and 253 K were simulated to be 50.3, 110, 167 and 277, respectively. The calculated results of the absolute rate constants were shown in Table S9.

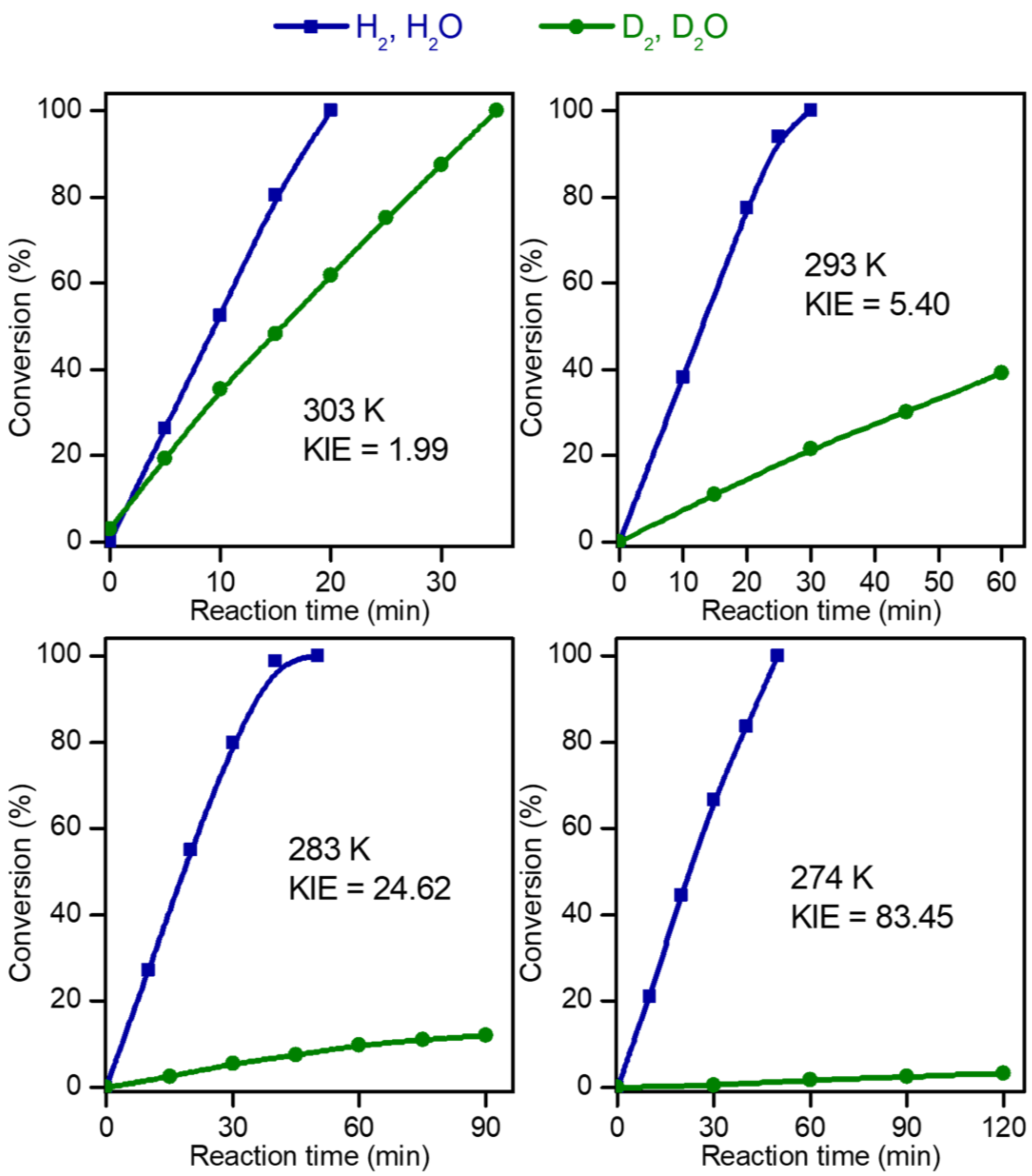

Figure S19. Experimentally measured KIE ($k_{H2,\ H2O}/k_{D2,\ D2O}$) at different temperatures. The KIE ($k_{H2,\ H2O}/k_{D2,\ D2O}$) values at 303, 293, 283 and 274 K were 1.99, 5.40, 24.62 and 83.45, respectively. Reaction conditions: 0.1 μmol Pd, 100 μmol benzaldehyde, 5 mL H_2O or D_2O, 0.1 MPa H_2 or D_2.

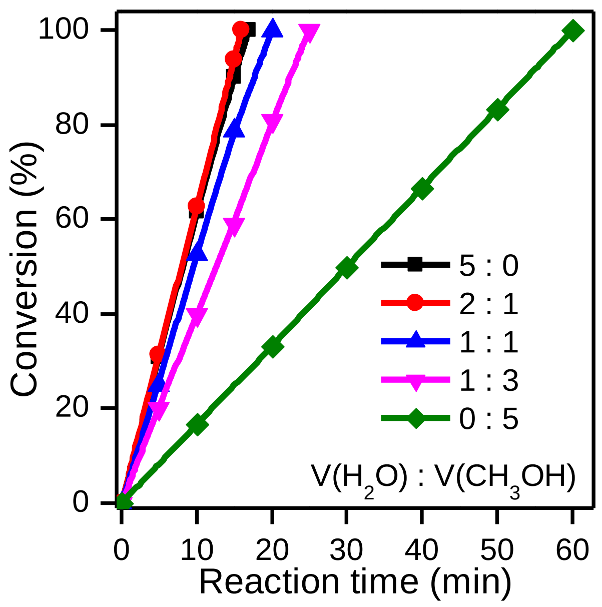

Figure S20. Catalytic performances of Pd_1/TiO_2 in 5 mL mixed solvent with different ratios of H_2O and CH_3OH. Adding H_2O to the reaction in CH_3OH increased the hydrogenation rate, reaching a level comparable to that of pure H_2O when the volume ratio of H_2O to CH_3OH was 2.

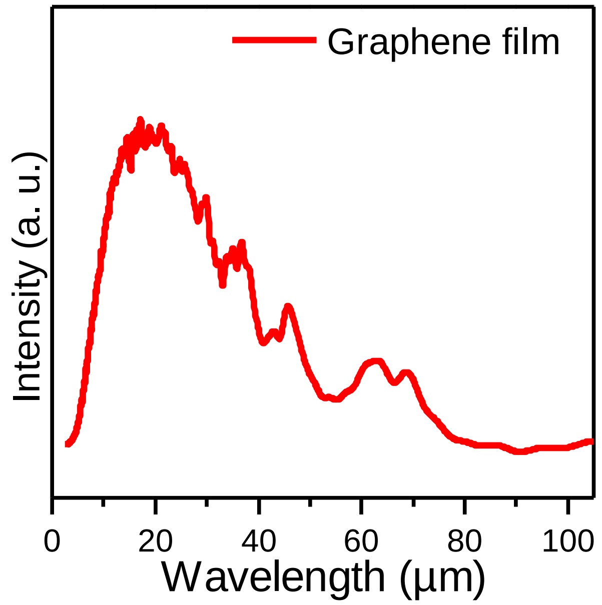

Figure S21. The far infrared irradiation with a broad distribution of 2.5-100 μm generated electrically by a flexible graphene film. The graphene film (XHGHF-C3) used in this work was obtained from Xiamen XiHe Technology co., Ltd.

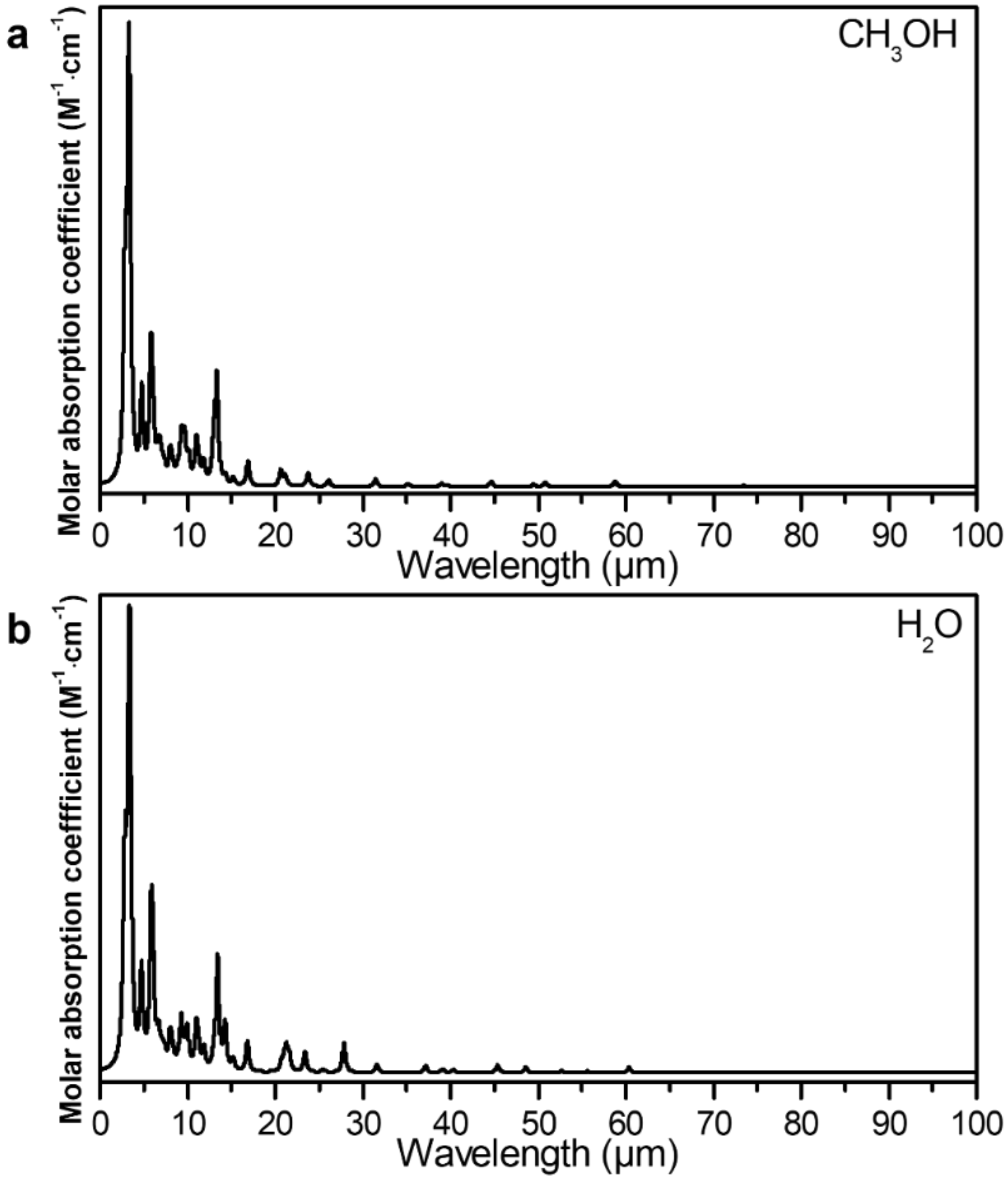

Figure S22. Simulated absorption region of hydrogen bond network formed in CH_3OH (a) and H_2O (b). The results are obtained from time-dependent density functional theory calculation based on optimized structures of electronic ground states at B3LYP/6-31G(d) level.

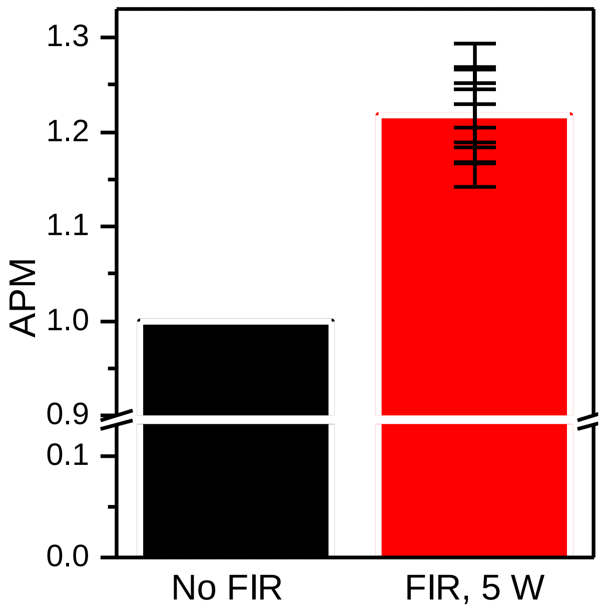

Figure S23. Catalytic performances of Pd_1/TiO_2 without or with FIR irradiation generated by graphene film in CH_3OH. In the benzaldehyde hydrogenation facilitated by FIR irradiation, the graphene film was electrically powered at 5 W. The reaction temperature was kept at 273 K by circulating cooling water to keep the temperature fluctuation at ±0.05°C.

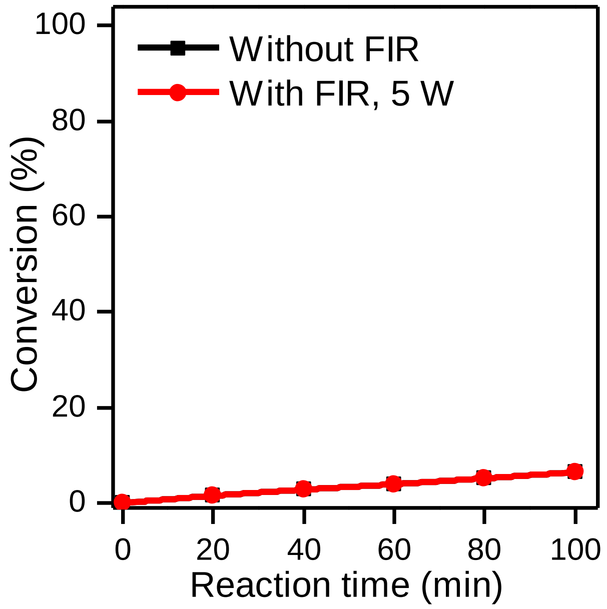

Figure S24. Catalytic performances of Pd/C without or with FIR in H_2O. In the benzaldehyde hydrogenation facilitated by FIR irradiation, the graphene film was electrically powered at 5 W. The reaction temperature was kept at 273 K by circulating cooling water to keep the temperature fluctuation at ±0.05°C.

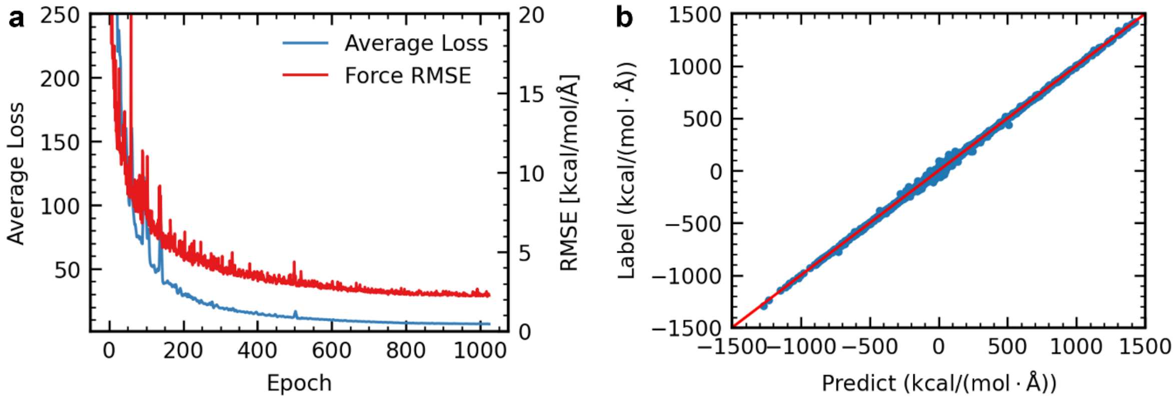

Figure S25. (a) Evolution of the loss function (blue line) on the training set and the root-mean-square error (RMSE) of predicted forces (red line) on the validation set, plotted against training epochs. The parallel decreasing trends of both curves indicate stable model optimization without overfitting, suggesting effective learning of the underlying physical interactions. (b) Scatter plot (blue dots) comparing the predicted forces (x-axis) against true forces (y-axis) across the entire dataset, with ideal agreement represented by the diagonal red line (slope = 1). The tight clustering of points around the ideal line validates the model's consistency across diverse atomic configurations.

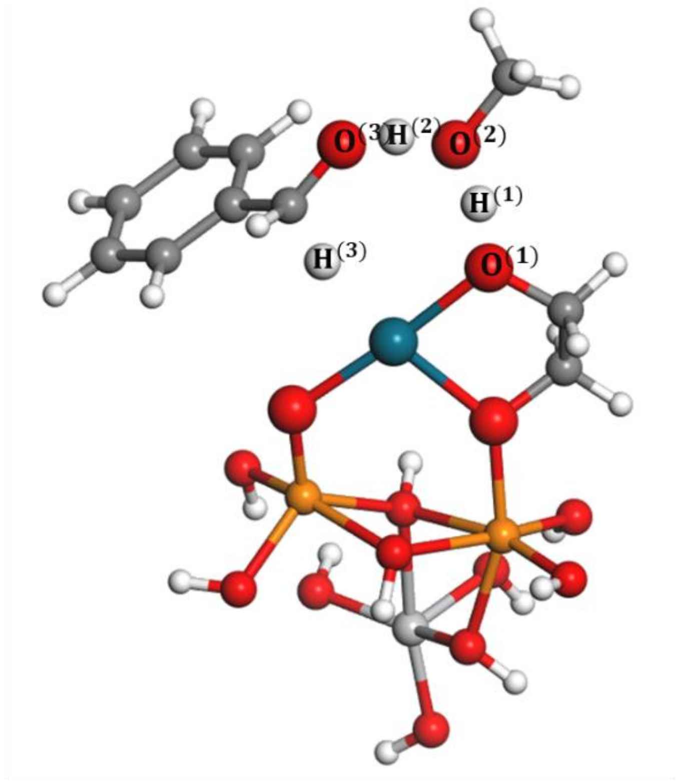

$$CV = d_{O^{(3)}H^{(2)}} + d_{O^{(2)}H^{(1)}} - d_{O^{(1)}H^{(1)}} - d_{O^{(2)}H^{(2)}}$$

Figure S26. Atomic structure of the simulated system. The structural model is the same as the one used in the DFT calculations. Turquoise, red, yellow, grey and white balls indicate Pd, O, Ti, C and H atoms, respectively. Collective variables (CVs) used in the enhanced sampling simulations is listed below the structure. The CVs were carefully selected to ensure proper sampling of the reaction pathway.

Table S1. Reaction scope of Pd_1/TiO_2 catalyzed hydrogenation of substituted benzaldehydes.

Substrates	Products	Time (min)	Conversion (%)	Selectivity (%)
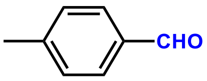	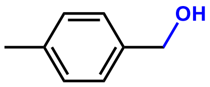	70	>98	>99
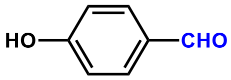	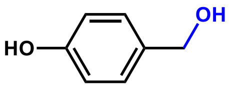	70	>99	>99
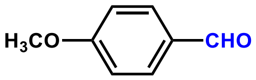	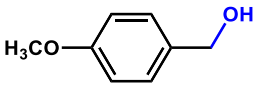	70	>99	>99
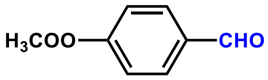	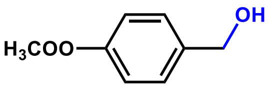	70	>99	>99
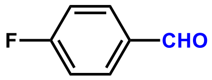	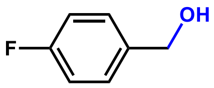	70	>98	>99
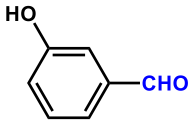	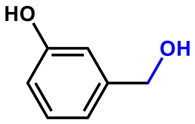	70	>99	>99
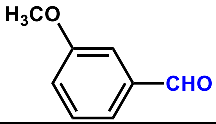	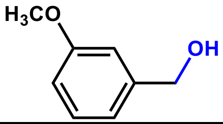	70	>98	>99

Reaction conditions: Pd_1/TiO_2 (0.1 μmol Pd), 5 mL CH_3OH, 0.1 MPa H_2, 303 K, substrate (100 μmol).

Table S2. Calculated KIE ($k_{H2, CH3OH}/k_{D2, CD3OD}$), reaction barrier and imaginary frequency of benzaldehyde (Ph-CHO) hydrogenation based on stepwise mechanisms.

Stepwise Mechanism 1	Ph-CHO to Ph-CHOH [a]	Ph-CHOH to Ph-CH_2OH [b]
Barrier (kJ/mol)	98.35	30.06
Frequency (cm^{-1})	578(H)/461(D)	343(H)/285(D)
KIE	2.33	46.38
**Stepwise Mechanism 2**	**Ph-CHO to Ph-CH_2O [c]**	**Ph-CH_2O to PhCH_2OH [d]**
Barrier (kJ/mol)	77.49	22.83
Frequency (cm^{-1})	643(H)/513(D)	1134(H)/823(D)
KIE	2.62	19.46

Stepwise Mechanism 1: $H^{\delta+}$ transfer followed by $H^{\delta-}$ transfer

[a] The transfer of $H^{\delta+}$ on O-H at the Pd-O interface to O atom of Ph-CHO, generating Ph-CHOH;

[b] The transfer of $H^{\delta-}$ on Pd-H at the Pd-O interface to C atom of Ph-CHOH, producing Ph-CH_2OH;

The KIE ($k_{H2, CH3OH}/k_{D2, CD3OD}$) value for the RDS of stepwise mechanism 1 is 2.33.

Stepwise Mechanism 2: $H^{\delta-}$ transfer followed by $H^{\delta+}$ transfer

[c] The transfer of $H^{\delta-}$ on Pd-H at the Pd-O interface to C atom of Ph-CHO, yielding Ph-CH_2O;

[d] The transfer of $H^{\delta+}$ on O-H at the Pd-O interface to O atom of Ph-CH_2O, producing Ph-CH_2OH.

The KIE ($k_{H2, CH3OH}/k_{D2, CD3OD}$) value for the RDS of stepwise mechanism 2 is 2.62.

Table S3. Calculated KIE values of k_{H2}/k_{D2}, k_{CH3OH}/k_{CD3OD} and $k_{H2,\ CH3OH}/k_{D2,\ CD3OD}$ of concerted triple H-tunneling mechanism at different reaction temperatures, B3LYP/6-311+G(d,p)/Lanl2dz level.

Temperature (K)	KIE		
	$k_{H2,\ CH3OH}/k_{D2,\ CD3OD}$	k_{H2}/k_{D2}	k_{CH3OH}/k_{CD3OD}
298	60.0	7.0	10.5
273	136	11.1	18.5
263	224	14.3	26.1
253	409	19.5	39.3
243	655	25.8	55.9
233	1239	36.3	89.9
223	2955	53.6	156

Table S4. Calculated absolute rate constants of benzaldehyde hydrogenation in CH_3OH at different temperatures after considering the quantum tunneling factor.

Temperature (K)	$k_{H2,\ CH3OH}$ (s^{-1})	$k_{D2,\ CD3OD}$ (s^{-1})	$k_{D2,\ CH3OH}$ (s^{-1})	$k_{H2,\ CD3OD}$ (s^{-1})
303	2.13×10^{-6}	3.95×10^{-8}	3.14×10^{-7}	2.13×10^{-7}
298	1.17×10^{-6}	1.95×10^{-8}	1.68×10^{-7}	1.12×10^{-7}
293	6.89×10^{-7}	9.83×10^{-9}	8.83×10^{-8}	5.79×10^{-8}
283	2.14×10^{-7}	2.23×10^{-9}	2.32×10^{-8}	1.46×10^{-8}
273	6.22×10^{-8}	4.59×10^{-10}	5.67×10^{-9}	3.38×10^{-9}
263	1.79×10^{-8}	8.48×10^{-11}	1.29×10^{-9}	7.20×10^{-10}
253	5.32×10^{-9}	1.40×10^{-11}	2.75×10^{-10}	1.41×10^{-10}

While there are discrepancies between the absolute values derived from simulations and those obtained from experimental results, the theoretical data are nonetheless capable of accounting for the experimental phenomena to a certain extent.

Table S5. Imaginary frequency of the transition structure for concerted triple H-tunneling mechanism in CH_3OH or H_2O, B3LYP/6-311+G(d,p)/Lanl2dz level.

	H_2+CH_3OH	D_2+CD_3OD	D_2+CH_3OH	H_2+CD_3OD
Frequency (cm^{-1})	1306	965	1159	1096
	H_2+H_2O	D_2+D_2O	D_2+H_2O	H_2+D_2O
Frequency (cm^{-1})	1320	975	1167	1112

Table S6. KIE values of k_{H2}/k_{D2}, k_{H2O}/k_{D2O} and $k_{H2, H2O}/k_{D2, D2O}$ of concerted triple H-tunneling mechanism at different reaction temperatures, B3LYP/6-311+G(d,p)/Lanl2dz level.

Temperature (K)	KIE		
	$k_{H2, H2O}/k_{D2, D2O}$	k_{H2}/k_{D2}	k_{H2O}/k_{D2O}
298	50.3	7.2	8.6
273	110	11.1	14.7
263	167	14.0	19.4
253	277	18.4	27.0

Table S7. Calculation results of reaction barrier, imaginary frequency of transition state, KIE ($k_{H2, CH3OH}/k_{D2, CD3OD}$) in concerted triple H-tunneling mechanism at 298 K.

Function	Barrier (kJ/mol)	Frequency (cm^{-1})	KIE
Cam-B3LYP	101.7	1312/969	46.9
M06	101.4	1192/897	35.6
B3LYP	112.9	1306/965	60.0

Table S8. Hyperparameters of GFN force field.

Feat. Dim.	Act. Func.	Cutoff	N_inter	N_heads	Readout_iter	Param Num
128	SiLU	0.6 nm	3	8	3	388609

N_epoch	Batch_size	Optimizer	Loss Func.	Learning rate	Metrics
1024	32	Adam	MSE	Exponential Decay	RMSE

Table S9. Calculated absolute rate constants after consider the quantum tunneling factor of benzaldehyde hydrogenation reaction in H_2O at different reaction temperatures.

Temperature (K)	$k_{H2, H2O}$ (s^{-1})	$k_{D2, D2O}$ (s^{-1})	$k_{D2, H2O}$ (s^{-1})	$k_{H2, D2O}$ (s^{-1})
298	9.56×10^{-4}	1.90×10^{-5}	1.33×10^{-4}	1.08×10^{-4}
273	1.04×10^{-4}	9.28×10^{-7}	9.18×10^{-6}	6.93×10^{-6}
263	4.01×10^{-5}	2.40×10^{-7}	2.86×10^{-6}	2.07×10^{-6}
253	1.57×10^{-5}	5.67×10^{-8}	8.51×10^{-7}	5.81×10^{-7}

While there are discrepancies between the absolute values derived from simulations and those obtained from experimental results, the theoretical data are nonetheless capable of accounting for the experimental phenomena to a certain extent.

Atomic Coordinates of Computational Models

1. H_2 activation process

Initial State

Ti	2.95335500	0.34626400	-0.62315800
O	2.40015400	1.40066000	-2.06673600
O	2.16586800	1.41967800	0.90054900
O	3.20041100	-1.32231100	-1.44251600
Ti	0.19593200	-1.78252500	0.44966400
Ti	0.07154400	1.50436700	0.72619400
O	0.72855500	-2.76860600	-1.04689200
O	0.19498000	-0.17214800	1.79506600
O	-1.49016600	-2.14219900	0.59985900
O	0.27884200	2.55765600	-0.75400700
O	-1.86391700	1.06456500	0.37143600
Pd	-2.70829200	-0.74614000	-0.33941600
C	-2.69943700	2.15145100	-0.11536300
O	-3.99912600	0.47859400	-1.20097000
C	-4.08909900	1.60724300	-0.35146900
H	-2.69130300	2.96150500	0.62583000
H	-2.27820100	2.52692600	-1.05571100
H	-4.56510800	1.35178000	0.61058900
H	-4.71997900	2.36603200	-0.84250300
O	4.64722000	1.03734400	-0.48515100
O	-0.17269000	2.75403200	2.05330900
H	5.36714700	0.40252900	-0.65638200
H	0.98838500	2.27382100	-1.38639300
H	3.09569000	2.00137500	-2.39011700
H	0.01681700	3.68007000	1.81606300
H	1.62634100	-2.45351900	-1.30246900
O	0.77320000	-0.11411900	-0.58048400
H	-0.50091300	-0.24349300	2.47241400
H	0.44730000	-0.10732200	-1.49725800
H	2.55840500	2.31126200	0.91222700

H	3.19581000	-1.21505800	-2.41259300
O	1.30092000	-2.66913400	1.74079400
O	3.31624500	-0.97728000	1.19614400
H	3.17240700	-0.33130100	1.91184400
H	2.64690700	-1.70345400	1.38557300
H	1.49599900	-3.58632700	1.48004900
H	-3.84624700	-2.17611700	-0.70562000
H	-3.39903900	-2.16063400	-1.35400100

Transition State

Ti	2.86643600	0.17062600	-0.53192500
O	2.35938100	1.15642700	-2.03234900
O	2.16499000	1.37180900	0.93590200
O	3.03385000	-1.55595000	-1.24826800
Ti	-0.00315500	-1.78005600	0.62255200
Ti	0.05984700	1.54062500	0.79391800
O	0.50635600	-2.83796700	-0.82906400
O	0.11004200	-0.12242400	1.90016300
O	-1.71513200	-2.08711100	0.73987600
O	0.33826100	2.54392500	-0.71404300
O	-1.85040300	1.23308800	0.46197000
Pd	-2.68033900	-0.62701000	-0.33215500
C	-2.71090700	2.25792500	-0.07193200
O	-3.88417700	0.62677100	-1.39898600
C	-4.07400300	1.68306100	-0.43930800
H	-2.83245500	3.04870300	0.68118300
H	-2.24411000	2.69492100	-0.96359700
H	-4.59193300	1.30810500	0.45249300
H	-4.69944000	2.45402500	-0.90991800
O	4.58585500	0.79573400	-0.41698400
O	-0.11374400	2.81666200	2.10863900
H	5.27768000	0.13951100	-0.62106700
H	1.02388700	2.19786500	-1.33793500
H	3.08703600	1.68490200	-2.40690100

H	0.26299100	3.69410000	1.91451400
H	1.42350000	-2.57463400	-1.07741800
O	0.65689400	-0.17820600	-0.46227600
H	-0.52971500	-0.13022800	2.63390600
H	0.33433700	-0.18308300	-1.38015900
H	2.59238900	2.24340800	0.85358000
H	3.03797500	-1.49965600	-2.22268900
O	1.05900900	-2.66729700	1.94371000
O	3.17521500	-1.08587000	1.35347100
H	3.04687800	-0.41349100	2.04722200
H	2.47113900	-1.77255400	1.54966800
H	1.19978100	-3.60422900	1.72027300
H	-4.31176400	-0.66930500	-1.20957300
H	-3.98088100	-1.58165300	-1.00952600

Final State

Ti	2.96053900	0.35927500	-0.60776200
O	2.41964800	1.40379600	-2.06325000
O	2.19661300	1.43156800	0.90247600
O	3.19878900	-1.31712600	-1.42551800
Ti	0.18031300	-1.79736700	0.42044600
Ti	0.04432200	1.50493300	0.71523400
O	0.72579300	-2.75553500	-1.08943500
O	0.18047400	-0.19470100	1.76449000
O	-1.49611400	-2.22340400	0.57231900
O	0.29551800	2.57838200	-0.75571200
O	-1.83332900	1.19118900	0.40428200
Pd	-2.69421100	-0.84427600	-0.32422500
C	-2.66909600	2.20569800	-0.15900000
O	-4.06003300	0.54276500	-1.16788300
C	-4.09130700	1.71527200	-0.29994700
H	-2.67237300	3.09361700	0.48830700
H	-2.28034300	2.50250400	-1.14199900
H	-4.51902000	1.43472900	0.66778600

H	-4.71317200	2.48100100	-0.77574600
O	4.66293300	1.03275100	-0.47513000
O	-0.13059600	2.75657800	2.06017800
H	5.37336800	0.40173700	-0.69295400
H	1.01094600	2.28357400	-1.37349800
H	3.11784600	2.00356800	-2.38208400
H	0.48605700	3.51012300	2.04670300
H	1.62269300	-2.43037400	-1.33690500
O	0.77090700	-0.10318500	-0.57828600
H	-0.45824500	-0.26822300	2.49499700
H	0.46023800	-0.08403100	-1.50020500
H	2.58998500	2.32252900	0.89939600
H	3.21581900	-1.20344800	-2.39459700
O	1.29201400	-2.69128900	1.69852400
O	3.31196800	-0.98146200	1.20996700
H	3.14456600	-0.34015800	1.92460500
H	2.64125500	-1.71092700	1.37468800
H	1.48765300	-3.60549100	1.42766300
H	-4.94740800	0.14001200	-1.18996700
H	-3.41422200	-2.05293200	-0.93854500

2. Triple H-tunneling mechanism in CH_3OH

2.1. One CH_3OH in model

Initial State

Ti	3.82303000	0.97851300	1.10332300
O	3.62681300	-0.11256300	2.59747000
O	4.01880300	-0.42083500	-0.31409000
O	3.00014400	2.58750400	1.61273600
Ti	0.77394300	1.30934200	-0.93777200
Ti	2.23226900	-1.62258600	-0.43236400
O	0.43987400	2.63090800	0.34148300
O	1.79998900	-0.28121200	-1.87178200
O	2.70183200	-2.30182400	1.19886600
O	5.61320500	1.33182800	1.32663200
O	2.97498500	-2.93154300	-1.51236500
H	5.83581200	2.27673000	1.41329600
H	3.05235000	-1.64824300	1.85410300
H	4.44352800	-0.22414400	3.11682400
H	2.72809500	-2.84498600	-2.45169300
H	1.27255000	2.79534100	0.84355100
O	1.75720400	0.27815800	0.57530700
H	2.60102100	0.02836400	-2.32773700
H	1.23092100	0.24162900	1.39318900
H	4.79053300	-0.97625200	-0.10017300
H	2.75076500	2.54406300	2.55539800
O	1.64315200	2.41836900	-2.23527100
O	4.01955700	2.25952800	-0.88372200
H	4.49115600	1.61605700	-1.44201700
H	3.16862800	2.42625700	-1.37846400
H	1.29246400	3.32606000	-2.22283700
O	-0.81879700	0.87248800	-1.45294300
O	0.38336900	-2.13552100	-0.40471100
O	-2.36935900	-2.84128800	-0.46045800
O	-5.60075600	-0.63588700	0.66435000
O	-3.48939200	-2.05473400	1.80212400

C	-0.08048700	-3.27642100	0.32302600
C	-1.32444300	-3.83983600	-0.33794000
C	-3.51564200	4.00711400	0.41842600
C	-4.71059100	4.00045000	-0.30710700
C	-5.47733800	2.83782300	-0.36226200
C	-5.04810700	1.67771800	0.30506500
C	-3.84643200	1.69244400	1.03790300
C	-3.08575500	2.85579400	1.09283700
C	-5.87244600	0.47063900	0.19708800
C	-4.20770800	-3.00329000	2.60347200
H	0.68706900	-4.06100500	0.34110200
H	-0.29885200	-2.98427500	1.35964400
H	-1.10421400	-4.16703100	-1.35754600
H	-1.71619900	-4.68657400	0.23684000
H	-2.91539300	4.91221400	0.46213400
H	-5.03994100	4.89632300	-0.82567300
H	-6.40785100	2.81819800	-0.92462700
H	-3.52434500	0.79665000	1.55924500
H	-2.15310500	2.87059700	1.64850200
H	-2.91041900	-0.39694800	-1.28773800
H	-6.81673800	0.59577300	-0.36336100
H	-4.71490400	-2.50134500	3.43639100
H	-3.47750800	-3.70961900	3.00649200
H	-4.94880200	-3.55251000	2.00922400
Pd	-1.52640800	-0.97349100	-0.96161100
H	-4.14888600	-1.43188400	1.41867300
H	-2.78755800	-2.65639500	0.43923300

Transition State

Ti	4.54819000	-0.55078400	-0.00125300
O	4.77201500	0.57909600	-1.46494200
O	3.71679700	0.72896900	1.31957600
O	4.44098300	-2.21575500	-0.84803200
Ti	1.05177900	-1.61704200	0.34426500

Ti	1.94577200	1.55848000	0.56795400
O	1.59171700	-2.88673800	-0.91893600
O	1.15369400	0.04108100	1.63455900
O	2.90450300	2.41776700	-0.72299500
O	6.25372100	-0.45979200	0.65318600
O	1.75142400	2.87059300	1.85294200
H	6.74994300	-1.29898900	0.63206300
H	3.62409500	1.88388700	-1.14897800
H	5.68123100	0.90882300	-1.58312300
H	1.17464900	2.62501000	2.59953500
H	2.57354200	-2.88101300	-0.97633500
O	2.33403000	-0.30836100	-0.54203000
H	1.68075000	-0.15537400	2.42732900
H	2.26090000	-0.28749900	-1.51221100
H	4.36287700	1.42262400	1.54773700
H	4.57925000	-2.12151400	-1.80930000
O	1.52719500	-2.59849000	1.91920800
O	4.14508600	-1.98203900	1.78035600
H	4.22511700	-1.39459200	2.55294000
H	3.19384000	-2.30138000	1.81753200
H	1.39639300	-3.55675500	1.80837900
O	-0.65388900	-1.50206400	0.02864400
O	0.19882300	1.65257500	-0.32141000
O	-2.37473300	1.96100300	-1.27863700
O	-4.47339100	-0.27662400	-2.32622600
O	-3.03254500	1.26988000	-3.49102200
C	-0.09599100	2.78253500	-1.17102200
C	-1.56616000	3.11431600	-1.02945000
C	-2.05290300	-4.56586700	-3.65879800
C	-2.25551300	-4.59630200	-2.27522200
C	-2.81818400	-3.49544600	-1.62881600
C	-3.18597700	-2.35562500	-2.35979600
C	-2.98648600	-2.33224000	-3.74688400
C	-2.42122400	-3.43301500	-4.39202200

C	-3.82038200	-1.19402400	-1.64151600
C	-3.81866300	2.24881400	-4.19410300
H	0.51524700	3.64312800	-0.87423200
H	0.14700300	2.52000200	-2.20944700
H	-1.76890500	3.47115800	-0.01073500
H	-1.84622900	3.90734100	-1.73681500
H	-1.61788900	-5.42416300	-4.16418000
H	-1.98149000	-5.47858300	-1.70250600
H	-2.98106000	-3.51888900	-0.55379800
H	-3.28326000	-1.45789300	-4.31795500
H	-2.27482300	-3.41094500	-5.46887000
H	-2.80875600	-0.64558700	-1.04383800
H	-4.32428100	-1.50670200	-0.71369900
H	-4.30270500	1.76506900	-5.04724800
H	-3.15426700	3.03868800	-4.55620200
H	-4.58274500	2.68516700	-3.53932100
Pd	-1.44455600	0.26622400	-0.62251300
H	-3.73250500	0.46387600	-3.04229800
H	-2.64187500	1.70018500	-2.49561600

Final State

Ti	-4.19176900	-0.08197200	-0.25587400
O	-4.43414100	-1.88692600	-0.64876900
O	-3.15645600	-0.29750500	1.48691400
O	-4.35743900	0.76178800	-1.91555000
Ti	-0.85098000	1.04038000	-1.15404000
Ti	-1.33062800	-1.28650900	1.12870500
O	-1.65771600	1.23380500	-2.82516600
O	-0.73159000	0.61751700	0.91492300
O	-2.28406700	-2.80841600	0.81149100
O	-5.82390800	0.17571400	0.53990000
O	-0.88518500	-1.54730900	2.89733200
H	-6.41202700	0.80494600	0.08312700
H	-3.09521700	-2.69334500	0.25389300

H	-5.31216400	-2.23259200	-0.40676400
H	-0.32536800	-0.84224500	3.27271800
H	-2.63475700	1.19963300	-2.70772600
O	-2.08579900	-0.57767100	-0.82802000
H	-1.28238300	1.23319800	1.42763000
H	-2.09647700	-1.20782900	-1.56966400
H	-3.69606000	-0.81786000	2.10971900
H	-4.64247700	0.12406700	-2.59716600
O	-1.17150600	2.82922000	-0.51911500
O	-3.64332300	2.08624200	0.30822800
H	-3.50702500	2.01032100	1.26960700
H	-2.77323300	2.44564600	-0.04033600
H	-1.20202700	3.47627500	-1.24500900
O	0.79807000	0.70851600	-1.55635900
O	0.35041400	-1.81075200	0.21440300
O	3.06330600	-2.00408400	0.47121900
O	3.65388900	0.43119100	-1.05101000
O	4.80528700	-1.90631100	-1.51681300
C	0.93195500	-3.13289000	0.32181400
C	2.24310300	-2.99665800	1.07311000
C	1.68030900	3.99183400	1.83768600
C	2.00279900	2.80602500	2.50237700
C	2.87764700	1.89052800	1.91151900
C	3.44179800	2.15215400	0.65546700
C	3.11728900	3.34782200	-0.00185800
C	2.23908100	4.26152500	0.58367900
C	4.35885500	1.14930900	0.01131500
C	6.15562400	-2.09350000	-1.08105000
H	0.23267000	-3.80090100	0.83818700
H	1.10221600	-3.51219000	-0.69413800
H	2.02887500	-2.73362100	2.12198800
H	2.77505200	-3.96101800	1.06959500
H	1.00311600	4.70744700	2.29711800
H	1.57837400	2.59515400	3.48074500

H	3.13019800	0.96832200	2.42970200
H	3.55826500	3.56351500	-0.97248300
H	1.99762500	5.18737500	0.06776700
H	4.72307000	0.42425300	0.74477600
H	5.21445000	1.63949500	-0.46541100
H	6.81379300	-1.67273700	-1.84652000
H	6.38671000	-3.16047700	-0.96704400
H	6.34967900	-1.58421200	-0.12786500
Pd	1.91435900	-0.62420900	-0.44929800
H	4.23943300	-0.33624900	-1.34473100
H	4.19000100	-2.18959200	-0.78065100

2.2. Two CH_3OH in model

Initial State

Ti	0.27680200	-3.10596100	-0.37833100
O	-1.09042100	-2.61059700	-1.54245900
O	1.88802200	-2.33104700	-1.31226300
O	-0.64412800	-3.35846800	1.23666300
Ti	1.37187100	-0.42041600	1.81640700
Ti	1.66915700	-0.24281900	-1.49001800
O	-0.12283100	-0.99144600	2.78952700
O	2.80898200	-0.31655200	0.24575100
O	0.32515500	-0.53130200	-2.70158300
O	0.47766800	-4.80798300	-1.03970200
O	3.07583400	0.04933500	-2.62509000
H	0.34104700	-5.52508600	-0.39398600
H	-0.31642200	-1.23723500	-2.43712100
H	-1.37716400	-3.31998400	-2.14531800
H	3.82839800	0.50135900	-2.15952600
H	-0.40744700	-1.86162000	2.42567700
O	0.34196300	-0.93008700	0.12438800
H	3.33399200	-1.13588100	0.23515800
H	-0.56081800	-0.53554500	0.12337000
H	2.00545600	-2.75915700	-2.17947500

H	-1.60739000	-3.33354600	1.08512800
O	2.65574900	-1.57455500	2.65260700
O	1.97624000	-3.68609000	1.12228500
H	2.75864300	-3.75055900	0.54657200
H	2.21298500	-2.96033300	1.77353100
H	2.42687700	-1.74570200	3.58306500
O	1.51959200	1.22562000	2.33417900
O	1.22832900	1.58334000	-1.02856300
O	-0.13116500	3.96381100	-0.29780200
O	-2.25803300	0.23970200	0.03047200
O	-2.60029200	3.02216200	-0.50956600
C	0.68412600	2.44215000	-2.03599900
C	0.61163000	3.87361800	-1.54117800
C	-7.27150400	-0.47060200	-0.53372200
C	-6.76027100	-1.45336400	0.31902300
C	-5.39962500	-1.46685100	0.61732600
C	-4.54680900	-0.49289800	0.06710200
C	-5.06812000	0.49292900	-0.79389000
C	-6.42653400	0.49982500	-1.08987700
C	-3.12668800	-0.56001200	0.40101300
C	-3.35456100	3.49939700	0.61684900
H	1.31230700	2.41658200	-2.93765100
H	-0.31964300	2.09297100	-2.31388200
H	1.60608500	4.27039100	-1.32231700
H	0.12429300	4.51071100	-2.28854400
H	-8.33224400	-0.45953100	-0.76968500
H	-7.41962300	-2.20350100	0.74575000
H	-4.98809300	-2.22613900	1.27800600
H	-4.40700400	1.23931100	-1.22178300
H	-6.83339000	1.25656600	-1.75445900
H	0.18570900	3.28123200	2.25080300
H	-2.82777500	-1.41189600	1.03304800
H	-4.34717100	3.03597000	0.64321800
H	-3.46896000	4.57923800	0.49375300

H	-2.83375600	3.29841200	1.56103500
Pd	0.66724000	2.51467500	1.00933300
H	-2.47321400	2.05426100	-0.39598800
H	-1.07966100	3.65239800	-0.44635200
O	4.79878100	1.24569100	-0.82245900
H	4.13497000	0.79439300	-0.24391000
C	6.06338800	0.60925700	-0.62144500
H	6.41460700	0.73651000	0.41125800
H	6.02135800	-0.46416500	-0.85250200
H	6.78199700	1.08387000	-1.29576300

Transition State

Ti	4.34494900	-0.56340000	0.06658700
O	4.70882600	0.36881000	-1.50374200
O	3.51925200	0.92894800	1.13943900
O	4.20856100	-2.31363400	-0.59106000
Ti	0.82920200	-1.50105600	0.35909000
Ti	1.79517600	1.67207500	0.21594600
O	1.39939000	-2.91943400	-0.70935700
O	0.95122400	0.26872400	1.50854400
O	2.84080800	2.37454600	-1.10417600
O	6.02259900	-0.49213400	0.79655800
O	1.50006900	3.11552700	1.29892800
H	6.46706100	-1.35162400	0.91478700
H	3.56306000	1.77213800	-1.41664000
H	5.63394500	0.65904900	-1.59884700
H	0.73375400	2.96499800	1.91464200
H	2.38441500	-2.92202100	-0.72629600
O	2.19821400	-0.32718300	-0.60288400
H	1.57018200	0.13167700	2.24703200
H	2.17082600	-0.43113100	-1.57038300
H	4.17745400	1.62993900	1.29986900
H	4.42522000	-2.33951800	-1.54182700
O	1.17137600	-2.34857700	2.05171900

O	3.77252100	-1.73823000	1.97118100
H	3.86073300	-1.09407500	2.69580700
H	2.81334700	-2.04004700	2.01828300
H	1.06749800	-3.31447100	1.99135000
O	-0.86187500	-1.40671700	-0.00846700
O	0.06575900	1.64264800	-0.71938100
O	-2.53953000	1.91422400	-1.66269000
O	-4.57141300	-0.52345600	-2.64848200
O	-3.28982300	1.15214700	-3.82053300
C	-0.23985100	2.67776100	-1.68121300
C	-1.69569700	3.06305300	-1.53640900
C	-1.53255500	-4.43135200	-3.90498000
C	-1.83992900	-4.53765000	-2.54481400
C	-2.58212400	-3.53657500	-1.91703900
C	-3.02368100	-2.41919800	-2.64205800
C	-2.72090700	-2.32262500	-4.00757400
C	-1.97745500	-3.32366900	-4.63410800
C	-3.84058000	-1.36373900	-1.94221900
C	-4.19145900	2.08941300	-4.43489500
H	0.39977300	3.55107600	-1.50509600
H	-0.03848300	2.29273600	-2.68984900
H	-1.86475300	3.51688400	-0.55099100
H	-1.97094300	3.79571000	-2.30783600
H	-0.95713100	-5.21168500	-4.39625800
H	-1.50761600	-5.40220600	-1.97600000
H	-2.82479300	-3.62072400	-0.86041800
H	-3.07108500	-1.46866400	-4.57860700
H	-1.75077500	-3.24335600	-5.69419600
H	-2.95826800	-0.69363900	-1.26695500
H	-4.35088600	-1.76042000	-1.05090200
H	-4.72579500	1.58637600	-5.24621600
H	-3.61150700	2.92026300	-4.84753200
H	-4.91548300	2.47638300	-3.70718400
Pd	-1.61969100	0.27193800	-0.86883500

H	-3.90866400	0.28151600	-3.35044100
H	-2.85741400	1.59750300	-2.84099000
O	-0.65012500	2.12418500	2.73321900
H	-0.24705000	1.31297300	2.33427100
C	-0.50777300	2.03522700	4.15403400
H	-1.06826400	1.18169200	4.55800400
H	0.54532500	1.94246200	4.45294400
H	-0.91403500	2.95520200	4.58400000

Final State

Ti	-4.56818700	-0.14452300	0.01761900
O	-4.75773800	-1.02945100	1.64233000
O	-3.39752500	1.39886700	0.66455700
O	-4.83770200	-1.48894600	-1.25996700
Ti	-1.33386800	-0.78235000	-1.56855100
Ti	-1.58178300	0.67032500	1.40677100
O	-2.21316200	-2.32191800	-2.14442100
O	-1.05496400	1.07318400	-0.54945400
O	-2.48912800	-0.05275600	2.80816600
O	-6.18662800	0.72202600	0.06872300
O	-0.97809900	2.21756400	2.15657600
H	-6.71825100	0.63171500	-0.74359000
H	-3.33847100	-0.50673800	2.56944400
H	-5.60456600	-0.87294200	2.09819000
H	-0.33262600	2.69614300	1.56962600
H	-3.17906500	-2.20634200	-1.99199600
O	-2.45612600	-0.90155000	0.14076800
H	-1.70978200	1.69909000	-0.90691400
H	-2.48841400	-1.80588300	0.49970000
H	-3.87478900	1.88065100	1.36478800
H	-5.16920400	-2.29566600	-0.82169300
O	-1.83656100	0.34998400	-3.03888600
O	-4.18151600	1.03199700	-1.90347300
H	-3.99343700	1.92383900	-1.56022300

H	-3.35049000	0.77711200	-2.40575700
H	-1.97143600	-0.15388700	-3.86022500
O	0.32995100	-1.24843400	-1.57565200
O	0.06592800	-0.45397200	1.47607600
O	2.77061200	-0.78011400	1.65421400
O	3.12993300	-1.60156800	-1.14821400
O	4.42390000	-2.67754300	0.90901000
C	0.62989300	-0.86440800	2.75023500
C	2.05358300	-0.35254800	2.80170300
C	5.63188800	2.69954000	-0.18229400
C	4.25540500	2.71214300	-0.42969900
C	3.64956500	1.62454400	-1.06092500
C	4.40786300	0.50824800	-1.44347000
C	5.78777100	0.50699500	-1.19674600
C	6.39831100	1.59704100	-0.57005300
C	3.74521500	-0.67792700	-2.09513400
C	5.77431500	-2.37447600	1.26876800
H	0.02203100	-0.45936300	3.56707000
H	0.60645900	-1.96099700	2.79744900
H	2.03677000	0.74834000	2.85893600
H	2.55528100	-0.73061800	3.70629600
H	6.10583200	3.54827600	0.30428700
H	3.65297800	3.56778000	-0.13610200
H	2.58095900	1.64119600	-1.25851600
H	6.38551300	-0.34919900	-1.50159600
H	7.46986300	1.58649100	-0.38767100
H	4.46694100	-1.26235500	-2.67490600
H	2.92971900	-0.37109000	-2.75286900
H	6.43349400	-2.91330800	0.58182400
H	5.99096100	-2.70560800	2.29314300
H	5.98305300	-1.29991000	1.19264400
Pd	1.52701600	-0.96359100	0.08110100
H	3.81060400	-1.97297900	-0.50868300
H	3.82041600	-2.07008700	1.42351300

O	0.66463400	3.12377800	0.13784800
H	0.21410000	2.34480300	-0.27113500
C	0.16237400	4.30377300	-0.49884100
H	0.40678000	4.31273000	-1.56890200
H	-0.92548000	4.39872900	-0.38045800
H	0.64237900	5.16262200	-0.02163900

3. Triple H-tunneling mechanism in H_2O

Initial State

Ti	-3.72455700	-1.09412700	1.07526400
O	-3.54062500	-0.03808300	2.59727900
O	-3.96524000	0.34763300	-0.28672100
O	-2.86276800	-2.69205800	1.55487500
Ti	-0.68267000	-1.27474200	-0.98164400
Ti	-2.22140100	1.59738800	-0.37639400
O	-0.30065300	-2.61492700	0.26909100
O	-1.73221500	0.31567400	-1.86220800
O	-2.71780400	2.22929700	1.26613000
O	-5.50842900	-1.47361800	1.29508700
O	-2.98123900	2.90629500	-1.44478100
H	-5.71817200	-2.42217800	1.37508400
H	-3.04552400	1.54574600	1.90327100
H	-4.35005500	0.02820100	3.13526000
H	-2.72815000	2.84361500	-2.38410000
H	-1.12136900	-2.82335400	0.77360000
O	-1.68970300	-0.30886000	0.56256600
H	-2.50126200	0.03187700	-2.38428100
H	-1.15922900	-0.27488500	1.37790500
H	-4.76368300	0.86691800	-0.08227100
H	-2.59948200	-2.66376900	2.49415700
O	-1.54293400	-2.38036800	-2.28763600
O	-3.90629300	-2.34459800	-0.92176200
H	-4.41730500	-1.72452400	-1.47161200
H	-3.05095700	-2.46173100	-1.42514800

H	-1.16430900	-3.27679800	-2.30233100
O	0.89511500	-0.79505700	-1.50432300
O	-0.38534900	2.15576800	-0.31302600
O	2.36671900	2.85445900	-0.18708700
O	5.76197300	1.05043900	1.53626600
O	3.24487800	2.10444600	2.18100000
C	0.03948800	3.27287700	0.47309700
C	1.31877900	3.85240500	-0.09943900
C	4.07232400	-3.48374700	-0.06283200
C	5.37339800	-3.28376800	-0.53423900
C	6.05857000	-2.11890100	-0.19380100
C	5.44106400	-1.14767200	0.61292000
C	4.13235600	-1.35693400	1.08727100
C	3.45339100	-2.52356100	0.74934400
C	6.19091600	0.07300800	0.92246700
H	-0.72784200	4.05808000	0.47221000
H	0.20015600	2.95015300	1.51111400
H	1.15861500	4.21088900	-1.11970400
H	1.67692500	4.68099600	0.52243200
H	3.53609300	-4.39171400	-0.32659600
H	5.84833300	-4.03234400	-1.16194500
H	7.07085900	-1.94995900	-0.55356300
H	3.66353700	-0.60808300	1.71769800
H	2.44041000	-2.68770300	1.10519400
H	2.95390900	0.47524700	-1.15219200
H	7.23325200	0.08857400	0.55558100
Pd	1.55479700	1.02415400	-0.85162500
H	4.09979700	1.65770800	1.97735100
H	2.71478900	2.64064300	0.73905800
H	3.49467300	2.86609300	2.73283000

Transition State

Ti	4.55034700	-0.55292400	0.01016300
O	4.78192500	0.57171700	-1.45539700

O	3.71639200	0.73231000	1.32378800
O	4.44364500	-2.22068400	-0.83191800
Ti	1.05367100	-1.61559400	0.35113600
Ti	1.94848200	1.56037300	0.56308300
O	1.59652300	-2.88987600	-0.90569700
O	1.15343500	0.04710400	1.63354000
O	2.91225700	2.41414700	-0.72731700
O	6.25386500	-0.46440100	0.67069600
O	1.75046900	2.87508800	1.84477000
H	6.74670200	-1.30574600	0.65919700
H	3.63298300	1.87881600	-1.14947100
H	5.69220000	0.89929300	-1.57147500
H	1.17107100	2.63203800	2.59014000
H	2.57850600	-2.88448900	-0.96145100
O	2.33999800	-0.31159200	-0.53730200
H	1.67618900	-0.14469900	2.43027500
H	2.26941900	-0.29546800	-1.50777300
H	4.36243900	1.42605500	1.55175200
H	4.58552900	-2.12941300	-1.79293800
O	1.51964000	-2.59125100	1.93211800
O	4.13940000	-1.98052600	1.79590700
H	4.21900500	-1.39105700	2.56700000
H	3.18772800	-2.29836600	1.83261100
H	1.38634400	-3.54953600	1.82431800
O	-0.65118900	-1.49946900	0.03076300
O	0.20473400	1.64893600	-0.33574400
O	-2.37307000	1.96094000	-1.27929700
O	-4.49370500	-0.28750000	-2.28556100
O	-3.05846000	1.24918900	-3.47843600
C	-0.08547900	2.76422200	-1.20590300
C	-1.55165000	3.11240300	-1.06151900
C	-2.04748400	-4.54134300	-3.69081400
C	-2.23007600	-4.58497500	-2.30483900
C	-2.79422700	-3.49499300	-1.64155400

C	-3.18341200	-2.35262600	-2.35741900
C	-3.00448700	-2.31636000	-3.74713900
C	-2.43758300	-3.40630400	-4.40917100
C	-3.81893200	-1.20383700	-1.61888200
H	0.53505500	3.62506900	-0.93017500
H	0.14854100	2.47877700	-2.24027000
H	-1.74431800	3.49267800	-0.04964000
H	-1.82957000	3.89213500	-1.78424700
H	-1.61119500	-5.39107500	-4.20940200
H	-1.93955800	-5.46913200	-1.74324800
H	-2.94183500	-3.52906100	-0.56464900
H	-3.31696000	-1.44049300	-4.30723400
H	-2.30682700	-3.37394900	-5.48778100
H	-2.80931300	-0.64935400	-1.03173700
H	-4.30765100	-1.53221100	-0.68852000
Pd	-1.44140400	0.26610400	-0.62311800
H	-3.77509300	0.45359900	-3.01175400
H	-2.66518700	1.69883100	-2.47575900
H	-3.63727400	1.91991800	-3.88603900

Final State

Ti	-4.09764700	0.45189500	-0.19101300
O	-4.61724700	-1.18242500	-0.90649300
O	-3.16234700	-0.19007400	1.47835300
O	-4.07365700	1.53917900	-1.72506400
Ti	-0.56993200	1.16575500	-0.90406400
Ti	-1.50794600	-1.37676400	0.97735000
O	-1.32564600	1.68598700	-2.52724900
O	-0.60283800	0.42886400	1.08684400
O	-2.64430200	-2.66924800	0.37736900
O	-5.67861900	0.92206900	0.60786900
O	-1.18745700	-1.97362000	2.69461300
H	-6.11874600	1.70950200	0.23895000
H	-3.41384300	-2.35164300	-0.15764700

H	-5.55007400	-1.41342500	-0.74476000
H	-0.56435300	-1.42596600	3.20732500
H	-2.30146800	1.76575400	-2.41401800
O	-2.04514900	-0.26137600	-0.80959300
H	-1.10381600	1.03171400	1.66274400
H	-2.14948300	-0.75797000	-1.64033400
H	-3.79246000	-0.69187700	2.02737400
H	-4.46135700	1.05471000	-2.47807800
O	-0.65175500	2.87542900	-0.03515200
O	-3.24384900	2.42734000	0.64577600
H	-3.16381400	2.26251700	1.60178300
H	-2.32124300	2.69735500	0.36211500
H	-0.53882600	3.60895700	-0.66430900
O	1.02164000	0.67552000	-1.36364100
O	0.16782200	-1.97503700	0.09116800
O	2.82734500	-2.58594400	0.28881700
O	3.78543300	-0.19558000	-1.07868500
O	4.57129000	-2.63541300	-1.70622400
C	0.55795600	-3.37201000	0.08428800
C	1.87039900	-3.48335900	0.83290000
C	3.12076700	4.03397300	1.49498200
C	2.98989700	2.86546100	2.24949500
C	3.49125400	1.65663500	1.75818500
C	4.13212400	1.60565400	0.51347700
C	4.26330200	2.78487100	-0.23503700
C	3.75871700	3.99193400	0.25035800
C	4.66223300	0.30588700	-0.02330700
H	-0.22987000	-3.97563300	0.55037900
H	0.67630700	-3.68824800	-0.96027400
H	1.69520500	-3.26164000	1.89855300
H	2.25429400	-4.51318300	0.76119200
H	2.73565500	4.97580500	1.87748000
H	2.50196400	2.89431000	3.22039900
H	3.39230300	0.74762600	2.34715400

H	4.76944800	2.75511600	-1.19740100
H	3.87109600	4.90075400	-0.33540500
H	4.73985500	-0.44956800	0.76474200
H	5.64650000	0.44320300	-0.48428500
Pd	1.90230300	-0.96455400	-0.47025200
H	4.21064300	-1.03751200	-1.44419200
H	3.92108000	-2.86133600	-0.97953100
H	5.44539200	-2.76897700	-1.30009800
